# Rational Design of Glycolipid‐Based Coacervates for Sequestration of Macromolecules and Enveloped Viruses

**DOI:** 10.1002/smsc.70399

**Published:** 2026-10-25

**Authors:** Nikesh Dewangan, Sandeep Yadav, Sarani Biswas, Indrani Das Jana, Ananya Ghosh, Priyaraj Pal, Arindam Mondal, Amirul Islam Mallick, Pradip Kumar Tarafdar

**Affiliations:** ^1^ Department of Chemical Sciences Indian Institute of Science Education and Research Kolkata Mohanpur India; ^2^ Department of Biological Sciences Indian Institute of Science Education and Research Kolkata Mohanpur India; ^3^ Department of Bioscience and Biotechnology Indian Institute of Technology Kharagpur Kharagpur India

**Keywords:** broad‐spectrum antiviral, coacervate–microorganism interaction, glycolipids, liquid–liquid phase separation, supramolecular trap

## Abstract

Molecular self‐assembly offers a powerful route to engineer sophisticated materials that mimic biological compartmentalization. Inspired by liquid–liquid phase separation (LLPS), which drives the formation of cellular membraneless organelles, we rationally designed glycolipid‐based coacervates by tuning the attractive and repulsive intermolecular interactions. The low‐molecular‐weight glycolipid‐based polyanionic amphiphiles self‐assemble into stable coacervate droplets in an aqueous medium at low micromolar concentrations. The glycolipid‐based self‐coacervates demonstrate the ability to sequester dyes, drug‐like molecules, proteins, and microorganisms, including influenza and lentiviruses. During the sequestration, the coacervates appear to mask the viral surface proteins, blocking viral attachment to host cell receptors and thereby preventing viral entry into host cells.

## Graphical abstract

The rational design of novel glycolipid‐based self‐coacervates by tuning attractive (pi–pi and H‐bonding) and repulsive (electrostatic) interactions is reported. The low‐molecular‐weight glycolipid‐based MWGSD‐A_4_ coacervates can encapsulate dyes, drugs, proteins, and pathogenic viruses. The sequestration of viruses within coacervates masks viral surface proteins, interferes with viral entry, and may serve as a broad‐spectrum antiviral strategy.

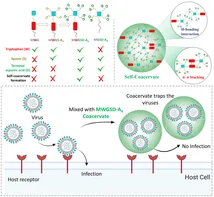

## 1 Introduction

The spatiotemporal orchestration of biochemical reactions within the dense, crowded milieu of the cytoplasm is largely governed by the fundamental principle of compartmentalization [1]. Traditionally, this organization was understood through membrane‐bound organelles; however, over the past decade, there has been a paradigm shift toward biomolecular condensates, membraneless organelles formed through phase separation [2]. Such condensates, often referred to as coacervates, are now recognized as ubiquitous and functionally indispensable, regulating diverse cellular processes, including signal transduction [3, 4], stress responses [5, 6], and sequestration of pathogenic components [7, 8]. An important feature of biomolecular condensates or coacervates is their ability to interact with a wide spectrum of molecular species, including small molecules [9, 10], proteins [11, 12], and membranes [13, 14, 15, 16]. Recently, synthetic coacervate‐based systems have emerged as powerful platforms for developing functional, life‐like biomaterials [17, 18, 19]. Coacervates are broadly classified into two types: simple and complex. In simple coacervation, or self‐coacervation, phase separation occurs from a single molecular component, such as a small molecule, peptide, or polymer. On the other hand, complex coacervation may result from interactions between oppositely charged macromolecules [20, 21, 22]. The high charge density and complex molecular structures of complex coacervates sometimes affect their biocompatibility, stability, and applicability in living systems [23, 24]. The emergence of self‐coacervates offers a transformative solution to these challenges [25, 26, 27, 28]. The self‐coacervation relies on a balance of attractive and repulsive intra‐ and intermolecular interactions, including hydrophobic interactions, electrostatic contributions, hydrogen bondings, cation–pi interactions, and solvation effects [10, 29, 30, 31, 32]. Recent studies have demonstrated that minimalist peptide synthons can undergo LLPS through a delicate balance of aromatic “stickers” and hydrated “spacers” [25, 33]. In addition, low‐molecular‐weight (LMW) compounds provide chemically defined, monodisperse platforms that enable precise tuning of intermolecular interactions and offer simplification of the molecular structure [23, 34]. These LMW systems exhibit rapid responsiveness to environmental stimuli and enhanced structural adaptability [35, 36]. In light of these findings, systematic studies on tuning the attractive and repulsive interactions and establishing the rational design principles of LMW self‐coacervates are essential. In addition, developing a single‐component LMW system that combines robust phase separation under physiological conditions with multifunctional therapeutic activity against pathogens remains an unresolved challenge [36, 37].

Current antiviral strategies largely rely on vaccine development [38] and small‐molecule inhibitors targeting viral enzymes [39, 40]. However, efforts to develop vaccines and virus‐targeting agents may be undermined by the rapid emergence of resistance, driven by mutations in the viral genome [41, 42]. Therefore, there is a critical need for active supramolecular materials capable of engaging viruses at the biointerface, sequestering virions, and neutralizing viral infectivity prior to cellular entry [43]. The integration of carbohydrate motifs into lipidated peptides [44, 45] and their interactions with the virus represent a promising yet underexplored strategy for virus–material interactions [46]. In this context, we reported the rational design of a glycolipid‐based scaffold and explored the roles of glucosamine, myristoylated tryptophan, succinyl linkers, and aspartic acid‐rich headgroups in self‐coacervation. Our studies suggest that among the glycolipid‐based amphiphiles, MWGSD‐A_4_ spontaneously forms LLPS under physiological conditions, yielding dynamic coacervates. These compartments exhibit modest sequestration of small molecules, proteins, and viruses. Taken together, the present report demonstrates the rational design of low‐molecular‐weight self‐coacervates and their potential to combat viral pathogens.

## 2 Results and Discussion

### 2.1 Design Principle of Glycolipid‐Based Coacervates

Glycolipids are indispensable architectural and recognition motifs in cellular membranes [47]. In addition, the fascinating phase behavior of glycolipids inspired researchers to investigate the lamellar and sponge phases. Mannose‐based [48, ‍^49^] and galactose/non‐ionic surfactant‐based glycolipids [50] were reported to form coacervates. These studies suggest that glycolipid‐based scaffolds could be an interesting motif for designing self‐ and complex coacervates. However, the self‐coacervation properties of various natural and synthetic glycolipids were not explored in detail. Here, we aim to rationally design low‐molecular‐weight self‐coacervates from glucose‐based glycolipids. Glucosamine‐based lipids were reported to form unilamellar structures [51]. To induce liquid–liquid phase separation, we invoked the sticker‐spacer model, originally described for amyloidogenic peptides, to carefully tune flexibility, H‐bonding, and π–π interactions [25]. Recently, the self‐assembly properties of GLYGGYGX suggested that when X is Trp (W), it induces coacervate‐like structures [52]. Therefore, we synthesized myristoyl tryptophan glucosamine (MWG) and characterized it by NMR and HRMS (Figures 1A, S1, S3, and S4). To identify the self‐assembly properties of the designed molecule, we investigated concentration‐dependent Nile Red (NR) fluorescence studies with MWG. The blue shift of NR emission revealed aggregation in an aqueous medium. The critical aggregation concentration (CAC) of MWG was estimated from the NR emission maxima (~50 µM, Figure 1B). Dynamic light scattering (DLS) studies suggested the presence of polydisperse large nanostructures ranging from 1 to 3 µm (Figure S14A). Confocal microscopic studies with NR as a probe indicate that the MWG aggregate didn’t have well‐defined structures (Figure 1F). They likely formed random aggregates due to associative (hydrophobic, H‐bond) interactions and were unable to sequester the hydrophilic dye, ruling out the possibility of coacervation (Figure ‍1J). To introduce a fine balance of associative and repulsive interactions, we incorporated succinate head groups into the design and synthesized myristoyl tryptophan glucosamine tetra succinic acid (MWGS‐A_4_) (Figures 1A and S1). The methylene linkers of succinate might serve as better spacers, and the electrostatic repulsion between carboxylates might promote coacervation. NR fluorescence revealed the self‐assembly of MWGS‐A_4_ with an estimated CAC of ~30 µM (Figure 1C). DLS studies with MWGS‐A_4_ suggested a polydisperse distribution centered at 1 µm (Figure S14B). Confocal microscopy with NR revealed that MWGS‐A_4_ adopted a spherical morphology (Figure 1G). However, the droplets were unable to encapsulate the hydrophilic dye (Figure 1K). These results show that modifying the design from MWG to MWGS‐A_4_ led to a change in morphology from random aggregates to a spherical morphology (emulsion or droplet). To further balance the attractive and repulsive forces, we incorporated more polyanionic groups (aspartic acids) at the head group and synthesized MWGSD‐A_4_ [10]. The CAC of MWGSD‐A_4_ was around ~30 µM (Figure 1D), and the size of the polydisperse solution was centered at around 1 µm (Figure S14C). Confocal microscopic studies with fluorophores revealed that the spherical droplets could encapsulate both hydrophobic and hydrophilic dyes, exhibiting coacervate‐like properties (Figure 1H,L). The DIC and cryo‐TEM images of MWGSD‐A_4_ further confirm the spherical nature of the coacervates (Figures S15 and S16). To check the importance of tryptophan and pi–pi stacking on coacervation, we synthesized myristoyl glucosamine succinate tetra‐aspartic acid (MGSD‐A_4_) with no tryptophan (Figures 1A and S2). The self‐assembly properties of MGSD‐A_4_ suggest that the CAC and aggregate size were ~40 and ~0.5 µm, respectively (Figures 1E and S14D). The spherical aggregates were capable of encapsulating hydrophobic (NR) dyes; however, they did not sequester hydrophilic dyes (Figure ‍1I,M). These results confirm the importance of the tryptophan moiety to adopt the coacervate structure [52]. Hence, we systematically explored the self‐assembly of glucose‐based lipids and rationally designed self‐coacervates from low‐molecular‐weight compounds by incorporating flexible linkers and balancing the supramolecular interactions (Figures 1A and 2F).

**FIGURE 1:**
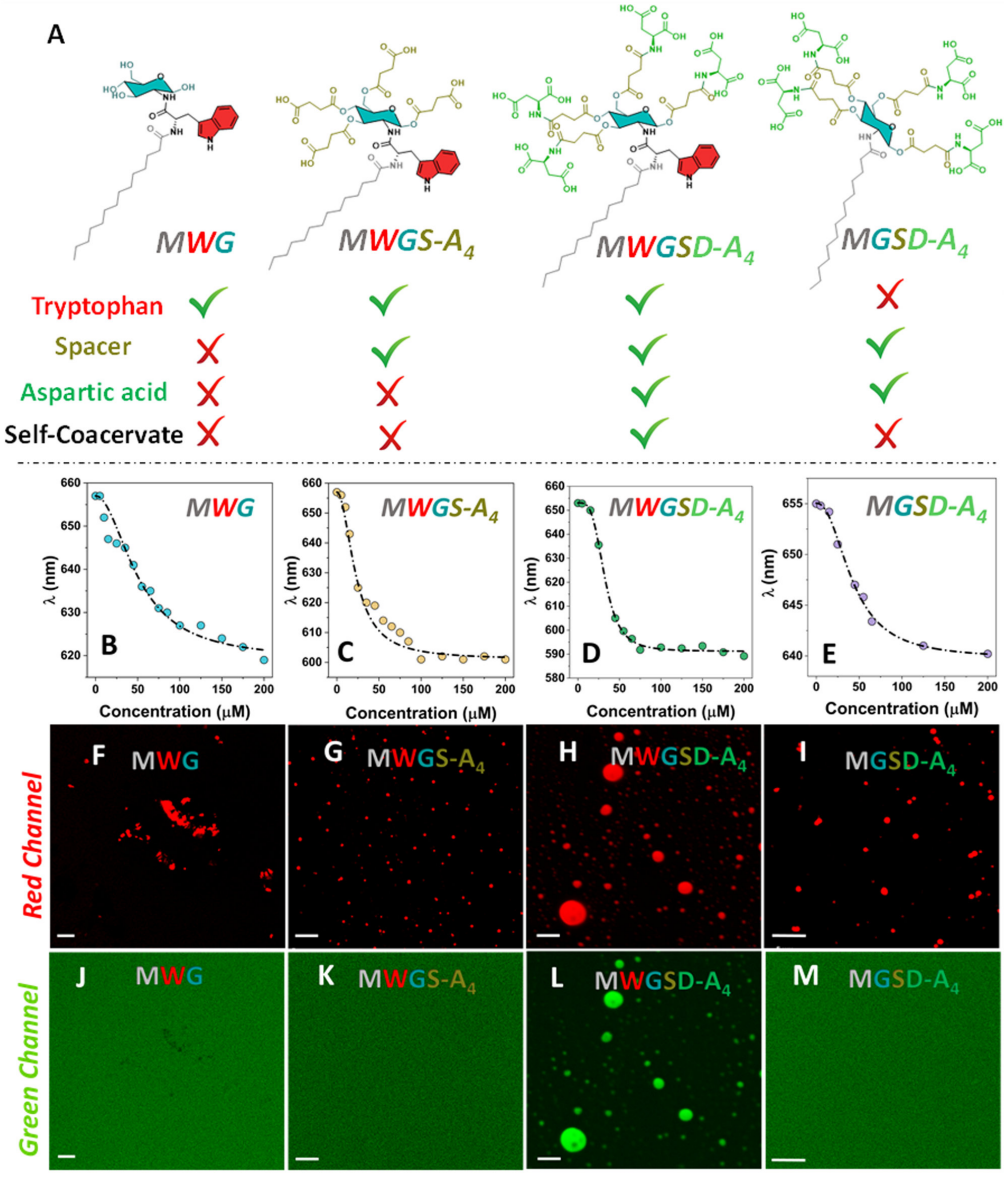
(A) Structure of the glycolipid‐based amphiphiles and self‐coacervate formation. (B–E) Concentration‐dependent NR emission maxima of MWG, MWGS‐A_4_, MWGSD‐A_4_, and MGSD‐A_4_. Dye encapsulation studies with Nile red (Hydrophobic) and fluorescein (hydrophilic): (F, J) MWG; (G, K) MWGS‐A_4_; (H, L) MWGSD‐A_4_; (I, M) MGSD‐A_4_. (All studies were performed at pH 5.5, 20 mM MES buffer.) The scale bar is 5 µm.

**FIGURE 2:**
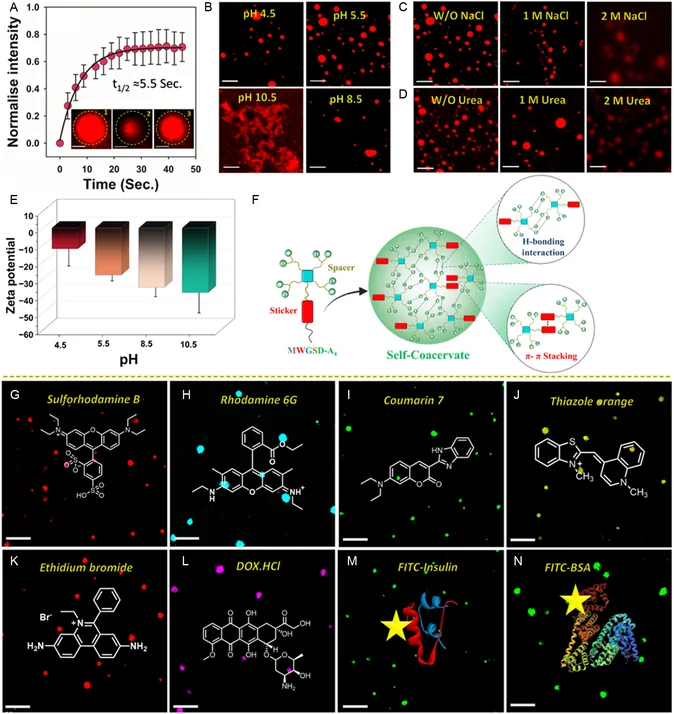
(A) FRAP measurement with sulforhodamine‐encapsulated MWGSD‐A_4_ compartment. Inset images show (1 = prebleach, 2 = bleach, 3 = recovery), scale bar 1 µm. (B) Coacervate stability at different pH. (C, D) Impact of NaCl and urea on self‐coacervation of MWGSD‐A_4_ (scale bar 2 µm). Nile red was used as the fluorophore for pH, NaCl, and urea‐dependent studies. (E) The zeta potential of the MWGSD‐A_4_ coacervates decreased with the increase in pH, suggesting that not all the ─CO_2_H of aspartic acids were deprotonated at pH 5.5. (F) Schematic representation of coacervate formation by MWGSD‐A_4_, illustrating the importance of H‐bonding and π–π stacking. (G–N) Small‐molecule fluorophores, anticancer drug doxorubicin, and protein sequestration in MWGSD‐A_4_ coacervates. Scale bar is 20 µm. Error bars represent the mean ± SD of at least three independent experiments.

### 2.2 Characterization, Robustness, and Versatile Encapsulation Ability of MWGSD‐A_4_ Coacervate

Encapsulation of hydrophilic and hydrophobic fluorophores suggests that MWGSD‐A_4_ forms coacervate‐like compartments. To further evaluate the dynamic, fluid‐like behavior of the self‐coacervates, we conducted fluorescence recovery after photobleaching (FRAP) experiments using sulforhodamine B (hydrophilic dye). The swift fluorescence recovery of MWGSD‐A_4_ (~80% in 50 s) confirms that the structures are coacervate droplets (Figure 2A) [53]. The MWGSD‐A_4_ droplets also exhibited co‐alescence, further emphasizing the fluidic nature of the compartment (Figure S17). Next, we evaluated the formation of MWGSD‐A_4_ coacervates at different pHs to understand the repulsive and COO^−^···HOOC hydrogen‐bonding interactions. The coacervates were found to be stable at a pH range of 4.5–8.5 (Figure 2B). However, the integrity of coacervates collapses into aggregates at higher pH, suggesting the critical balance between COO^−^···COO^−^ repulsive and COO^−^···HOOC hydrogen‐bonding interactions (Figure 2B). The zeta potential of the coacervates gradually increased as the pH increased (Figure 2E). We speculate that at the optimum pH level (4.5–8.5), not all the carboxylates get deprotonated; therefore, the partially deprotonated ─COO^−^ could form H‐bond with the undissociated ─CO_2_H to assist associative interactions, whereas repulsive interactions between partially deprotonated carboxylates are also essential to fine‐tune the balance between associative and repulsive interactions to stabilize the coacervate structure. At a higher pH of 10.5, deprotonation might disturb the fine balance and lead to aggregate formation (Figure 2B). Next, we evaluated the robustness of the coacervates under high ionic strength by increasing the NaCl concentration. MWGSD‐A_4_ maintained stable coacervate formation up to 1 M NaCl but destabilized at 2 M NaCl (Figure 2C). These results suggest that electrostatic repulsion between free carboxylates likely stabilizes the coacervate structures. The influence of hydrogen bonding was probed by investigating the coacervate stability in the presence of urea, a known H‐bond disruptor. The coacervates were stable at 1 M urea; however, at elevated urea concentrations, the coacervates became unstable, emphasizing the importance of hydrogen‐bonding interactions (Figure 2D,F). Time‐dependent confocal experiments suggest that the MWGSD‐A_4_ coacervates were stable for at least 24 h, indicating their robustness (Figure S18).

The low‐molecular‐weight self‐coacervate (MWGSD‐A_4_) exhibited efficient sequestration of a diverse set of hydrophilic and hydrophobic molecules (Figure 2G–K). The internal microenvironment of MWGSD‐A_4_, with a hydrophobic myristoyl chain and a hydrophilic aspartate, might be composed of well‐balanced fluctuating domains with a lower dielectric constant than that of water. As a result, dyes with a wide range of polarities were sequestered via hydrophobic stabilization, hydrogen bonding, and electrostatic interactions [54]. Beyond model dyes, the efficient encapsulation of the anticancer agent doxorubicin (Figure 2L) highlights its potential as a drug‐delivery platform. Recently, coacervate droplets have also emerged as model protocells and membraneless compartments due to their dynamic behavior and effective sequestration of biomacromolecules [55, 56]. We examined the protein encapsulation ability of MWGSD‐A_4_ coacervates using FITC‐labeled insulin and FITC‐labeled BSA. Confocal microscopy suggests that both insulin and BSA were encapsulated into the MWGSD‐A_4_ coacervates (Figure 2M,N). These findings demonstrate the diverse encapsulation behavior of MWGSD‐A_4_ coacervates, underscoring their promise for applications in drug delivery and biomimetic compartmentalization.

### 2.3 Virus Encapsulation and Clustering

Peptide‐based coacervates were reported to encapsulate nonenveloped viruses [43], and these studies emphasized that coacervate‐based formulations could help to thermostabilize viruses, facilitating vaccine storage. Given that our glycolipid‐based MWGSD‐A_4_ coacervate is robust in encapsulating both small molecules and macromolecules, we were curious whether small‐molecule‐based self‐coacervates could be useful for sequestering viruses. To test, we incubated GFP‐expressing lentivirus at 37 °C for 12 h in the presence of MWGSD‐A_4_ coacervate (Figure ‍3A). Notably, after incubation, the initially clear solution turned visibly turbid (Figure 3B), whereas control samples containing only GFP‐lentivirus remained clear. This qualitative shift suggests that coacervate likely interacts with the virus and promotes aggregation. The OD (scattering at 400 nm) of GFP‐lentivirus encapsulated in the MWGSD‐A_4_ coacervate was increased; however, the only lentivirus sample exhibited no change in the OD (Figure 3C). To visualize viral localization within the coacervate, we performed confocal microscopy experiments. GFP‐lentivirus particles after 12 h showed a very weak fluorescence signal (Figure ‍3D). However, the coacervate incubated with the virus showed a strong fluorescence signal (green). When NR was added to the solution, we observed colocalization of NR (red) and GFP (green) fluorescence. The merged images confirmed the localization of the GFP‐lentivirus inside the coacervate droplets (Figure 3E–G). The intensity plot of two fluorophores (dashed line) suggests that the concentrated virus is located within the coacervate (Figure 3G, inset). This colocalization indicates that MWGSD‐A_4_ coacervates could sequester viral particles during incubation.

**FIGURE 3:**
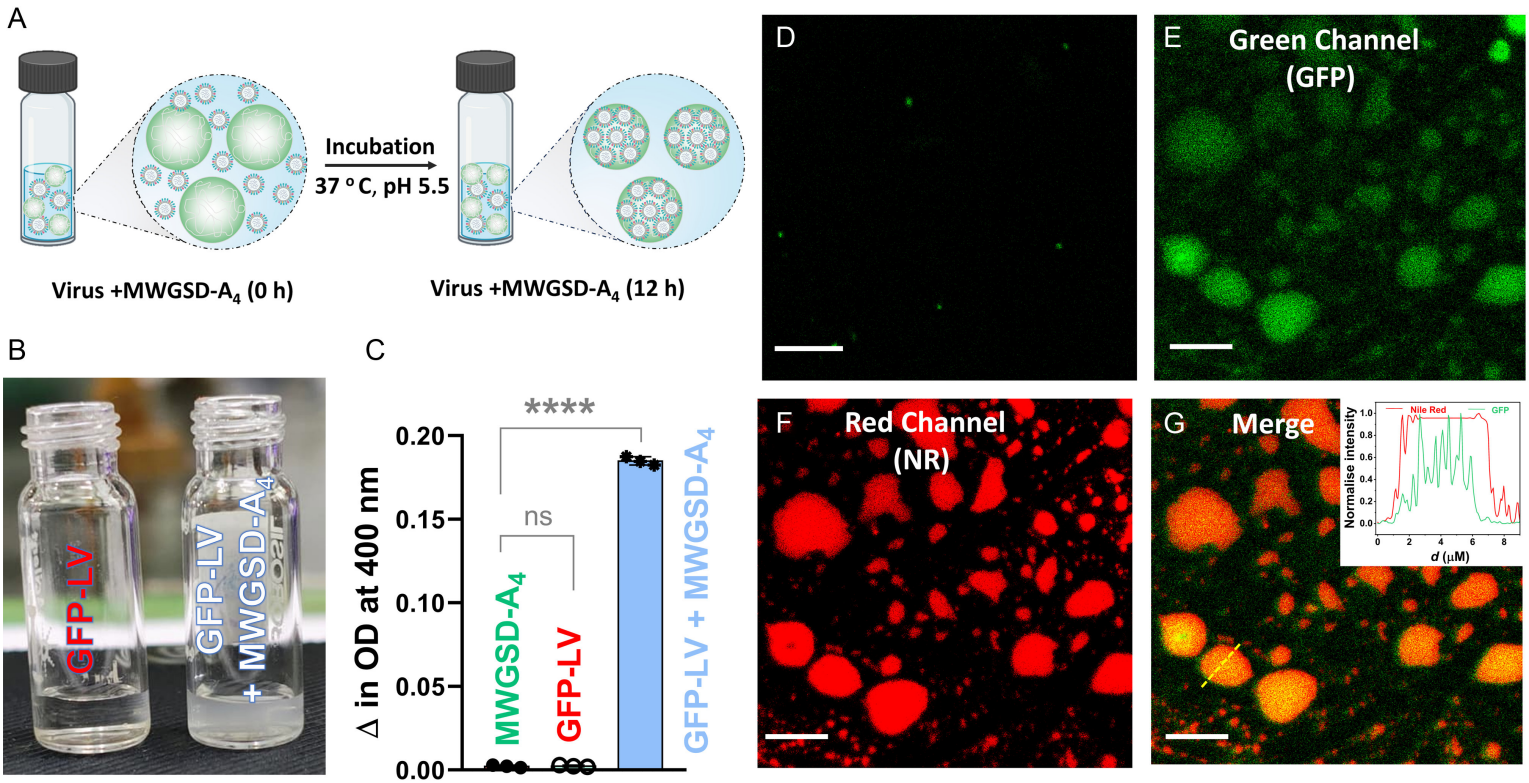
(A) Illustrates the sequestration of viruses in MWGSD‐A_4_ coacervate. (B) Turbidity of GFP‐lentivirus encapsulated coacervate increased with time. (C) The increase in OD (scattering at 400 nm) of GFP‐lentivirus encapsulated coacervate supports the virus sequestration. Experiments were repeated at least 3 times (*n* = 3). The results were compared by performing a two‐tailed Student’s *t*‐test for the experimental sets. Significance was denoted as follows: ns > 0.05 and ****p  ≤ 0.0001. Confocal image of (D) GFP‐lentivirus and (E) GFP‐lentivirus encapsulated in coacervate (Green). The lentivirus was incubated with coacervate for 12 h. (F) NR encapsulated coacervate to track the coacervate (red). (G) Merged image (yellow) demonstrates the localization of the virus in the coacervate. The inset figure shows the intensity plot of the merged image, green: GFP‐lentivirus; red: NR. The scale bar is 10 µm.

Next, we examined whether the MWGSD‐A_4_ coacervate exhibits a similar effect on the influenza virus. The virus was incubated with MWGSD‐A_4_ for 12 h under conditions similar to those described earlier. The solution became turbid after incubation, whereas control samples containing only MWGSD‐A_4_ or only the influenza virus remained clear (Figure S19). Consistent with previous observations, the OD at 400 nm showed that the influenza virus–MWGSD‐A_4_ mixture exhibited a pronounced increase relative to both control solutions (Figure 4A). This result indicates that the MWGSD‐A_4_ coacervate induces influenza viral clustering in a manner analogous to its effect on GFP‐lentivirus. Unlike GFP‐lentivirus, the influenza virus used herein lacks an intrinsic fluorophore. Therefore, we employed cryo‐TEM to visualize the viral aggregation. Cryo‐TEM images of the influenza virus showed a uniformly dispersed viral population (inset, Figure 4B), whereas samples incubated with MWGSD‐A_4_ showed potential viral clustering (Figure 4B).

**FIGURE 4:**
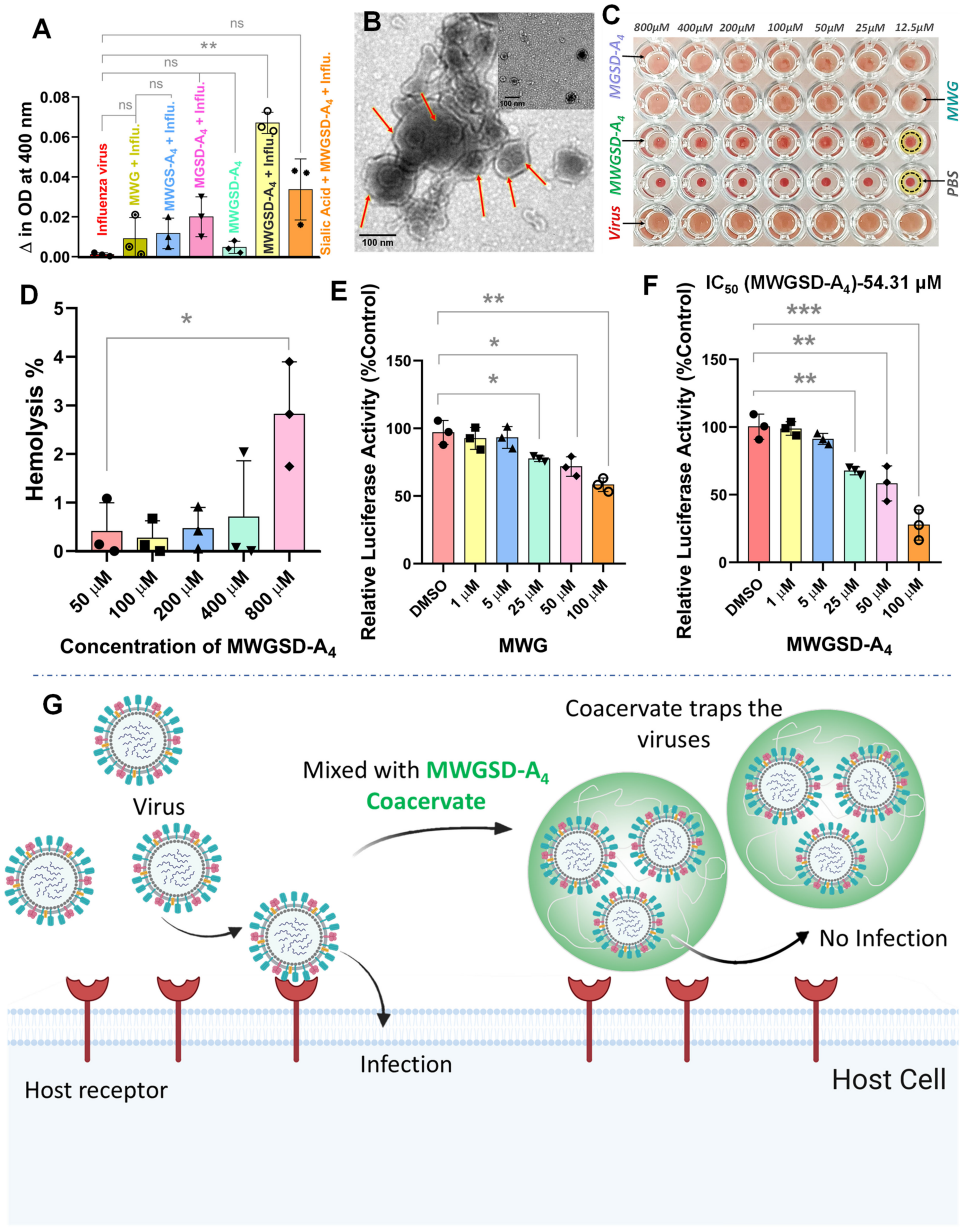
(A) Change in OD (scattering at 400 nm) of influenza virus incubated with designed glycolipids. MWGSD‐A_4_ coacervate exhibited a significant increase in OD. The virus was also preincubated with 1 mM sialic acid, and the experiment was performed in the presence of MWGSD‐A_4_. Experiments were repeated at least 3 times (*n* = 3). (B) Cryo‐TEM image shows the encapsulation of the influenza virus within the coacervate (inset: only virus, which appears to be dispersed). (C) Hemagglutination inhibition with designed glycolipids. (D) Hemolysis of RBCs at the various concentrations of MWGSD‐A_4_. Experiments were repeated at least 3 times (*n* = 3). Antiviral efficacy of (E) MWG and (F) MWGSD‐A_4_ against the infectious reporter Influenza A (H1N1) virus in MDCK cells. Experiments were performed in triplicate and repeated at least 3 times independently. (G) Schematic representation of how MWGSD‐A_4_ coacervate facilitates the trapping of viral particles and prevents viral entry into the host cell. Results were compared by performing a two‐tailed Student’s *t*‐test for the experimental sets. Significance was denoted as follows: ns > 0.05; *p ≤ 0.05; **p ≤ 0.01; and ***p ≤ 0.001. Graphs were plotted using GraphPad Prism 10.1.2.

Further, we performed turbidimetric virus‐sequestering experiments with MWG, MWGS‐A_4_, and MGSD‐A_4_ to evaluate the role of different compartments. MWG, which forms aggregates rather than droplets, did not produce a significant increase in OD, indicating that MWG did not promote viral sequestration (Figure 4A). Among the spherical emulsion‐like assemblies, MGSD‐A_4_ with tetra‐aspartic acid (more anionic) showed a slight increase in turbidity, whereas a negligible increase in turbidity was observed with MWGS‐A_4_ (tetra succinate) (Figures 1G,I and 4A). However, the increase in OD was substantially lower (~3.5‐fold) than that observed for MWGSD‐A_4_ coacervates (Figure 4A). These results indicate that the polyanionic aspartate‐rich emulsion‐like droplets (MGSD‐A_4_) can promote some degree of virus sequestration, but the coacervate nature of MWGSD‐A_4_ significantly enhances viral clustering (Figures 1A and 4A). From these data, we speculate that the polyanionic interface (tetra‐aspartate) could support viral sequestration through multivalent electrostatic interactions with the cationic patches on viral surface proteins. An important question now is whether coacervates sequester the virus via nonspecific interactions or via specific binding to the glycan motif. Therefore, the influenza virus was preincubated with 1 mM sialic acid and then treated with MWGSD‐A_4_. The increase in OD suggested virus sequestration in the presence of sialic acid (Figure 4A). However, the extent of the increase was much lower than that observed in the absence of sialic acid (Figure 4A). It appears that sialic acid could hamper the virus‐MWGSD‐A_4_ interaction, but was unable to inhibit it. These observations suggest that a polyanionic MWGSD‐A_4_ coacervate could support viral clustering, but specific binding to viral protein and MWGSD‐A_4_ cannot be ruled out. Further studies are required to understand the nature of virus–MWGSD‐A_4_ interactions.

### 2.4 Inhibition of Hemagglutination and Antiviral Defense

The encapsulation and clustering of the influenza virus motivated us to examine MWGSD‐A_4_’s ability to interfere with the interaction between the influenza hemagglutinin (HA) protein and RBC surface sialic acids. We performed a hemagglutination‐inhibition assay (HAI) using the A/Puerto Rico/8/1934 (H1N1) strain at an MOI of 0.1. In the virus‐only control, RBCs were fully agglutinated (Figure 4C). The addition of MWGSD‐A_4_ resulted in the inhibition of hemagglutination, as evidenced by the formation of a compact RBC button. The other glycolipid‐based designs (MWG and MGSD‐A_4_), which didn’t form coacervates, exhibited no hemagglutination inhibition (Figure 4C). These observations highlight the ability of MWGSD‐A_4_ coacervates to interfere with HA‐sialic acid interactions and suggest their potential importance in combating influenza infection.

Cytocompatibility studies of MWGSD‐A_4_ in MDCK cells showed no detectable cytotoxicity up to 200 µM (Figure S20). We also assessed the potential hemolytic activity of MWGSD‐A_4_. Our results revealed that MWGSD‐A_4_ exhibits negligible or no hemolytic activity even at high concentrations (800 µM) (Figure 4D). Next, we employed an infectious reporter influenza A/WSN/1933 (H1N1) strain, engineered to express nanoluciferase (PASTN), allowing quantitative monitoring of viral infection in MDCK cells [57]. As shown in Figure 4F, MWGSD‐A_4_ significantly inhibited viral entry in a dose‐dependent manner, with an IC_50_ value of 54.3 µM (Figure 4F). Similar studies were performed with MWG as a noncoacervate‐forming assembly (Figure 4E). We found that MWG couldn’t inhibit viral infection as effectively as MWGSD‐A_4_ (Figure 4E,F). The influenza virus‐trapping ability of MWGSD‐A_4_ coacervates might mask the influenza hemagglutinin, thereby interfering with viral attachment to host cell surface receptors and inhibiting viral entry (Figure 4G). The sequestration of microorganisms advocates the promising potential of glycolipid‐based coacervates for combating other viral infections and for vaccine storage.

The systematic comparison of the phase behavior of MWG, MWGS‐A_4_, MWGSD‐A_4_, and MGSD‐A_4_ established a rational molecular design for the development of low‐molecular‐weight glycolipid‐based self‐coacervates. MWG with a myristoyl chain, tryptophan, and glucosamine forms irregular aggregates (Figure 1A,F,J), whereas the introduction of succinyl groups (MWGS‐A_4_) leads to emulsion‐like droplets (Figure 1A,G,K). Further incorporation of four aspartic acids into the headgroup (MWGSD‐A_4_) leads to the formation of coacervate droplets (Figure 1A,H,L). However, a similar headgroup structure with aspartate but no tryptophan (MGSD‐A_4_) did not form coacervates (Figure 1A,I,M). This highlights the importance of aromatic moiety (tryptophan) and pi–pi interactions in stabilizing the dense liquid phase. The molecular structure of MWGSD‐A_4_ provides an appropriate balance of hydration, hydrogen bonding, molecular flexibility, hydrophobic interactions, and electrostatic repulsion to stabilize the LLPS. The tetra‐aspartic acid headgroup of MWGSD‐A_4_ coacervate generated a polyanionic interface (negative zeta potential; Figure 2E), which initiated viral sequestration through multivalent electrostatic interactions with the cationic patches on viral surface proteins. The viral sequestration likely inhibited viral entry into cells, thereby combating viral infection (Figure 4F). In general, polyanionic antiviral systems, including carrageenan and heparan sulfate mimetics, are shown to exhibit potent antiviral activity through interactions with viral proteins [58, 59]. However, these polyanionic materials also raised concerns about off‐target effects, for example, nonspecific interactions with blood coagulation proteins, thereby limiting their in vivo applications [60, 61, 62]. Although MWGSD‐A_4_ is a low‐molecular‐weight compound compared with heparin mimetics, its polyanionic character warrants similar caution in biological applications. While MWGSD‐A_4_ showed negligible hemolysis and good compatibility with mammalian cells under the tested conditions, further studies on serum‐protein adsorption, coagulation compatibility, and in vivo safety are needed for translational applications. Together, the present study demonstrated the fundamental materials science of rationally designed low‐molecular‐weight self‐coacervates and their potential as a supramolecular platform for virus sequestration.

## 3 Conclusion

The phase behavior of glycolipid mimics was studied, and a rational design principle of glycolipid‐based self‐coacervation was proposed. By careful modulation of the attractive (pi–pi and H‐bonding) and repulsive (electrostatic) interactions, we reported the self‐coacervation of MWGSD‐A_4_ at relatively low concentrations (~30 µM). MWGSD‐A_4_ coacervates were stable over a pH range of 4.5–8.5 and exhibited a balanced dynamic microenvironment. The low‐molecular‐weight self‐coacervates efficiently encapsulated a variety of small molecules with diverse polarity, drug‐like compounds, and proteins. Beyond proteins, MWGSD‐A_4_ could also encapsulate biological entities, including microorganisms such as influenza viruses and lentiviruses. The coacervate induced viral sequestration and masked the influenza receptor‐binding protein, thereby interfering with hemagglutination. Antiviral assays using a bioluminescent reporter influenza A virus (PASTN) revealed that MWGSD‐A_4_ significantly inhibits viral entry, with an IC_50_ of 54.3 µM. Collectively, our results established the rational design of glycolipid‐based self‐coacervates and highlighted the promising potential of coacervate engineering for developing broad‐spectrum antivirals.

## Author Contributions

Conceptualization: **Nikesh Dewangan** and **Pradip Kumar Tarafdar**. Methodology: **Nikesh Dewangan**, **Sandeep Yadav**, **Indrani Das Jana**, and **Sarani Biswas**. Investigation: **Nikesh Dewangan**, **Sandeep Yadav**, **Sarani Biswas**, **Indrani Das Jana**, **Ananya Ghosh**, and **Priyaraj Pal**. Supervision: **Arindam Mondal**, **Amirul Islam Mallick**, and **Pradip Kumar Tarafdar**. Funding acquisition: **Arindam Mondal**, **Amirul Islam Mallick**, and **Pradip Kumar Tarafdar**. Writing original draft: **Nikesh Dewangan** and **Pradip Kumar Tarafdar**. Writing review and editing: **Nikesh Dewangan**, **Sandeep Yadav**, **Indrani Das Jana**, **Arindam Mondal**, **Amirul Islam Mallick**, and **Pradip Kumar Tarafdar**.

## Funding

PKT sincerely thanks the ANRF/Science and Engineering Research Board (SERB‐DST) Core Research Grant (Grant No: CRG/2022/008554) for the financial support. AM acknowledges the Core Research Grant (CRG) from ANRF/Science and Engineering Research Board (SERB‐DST) (Grant no: CRG/2022/003628); Research Grant from West Bengal Science & Technology and Biotechnology Department (Memo No. 1111(SANC.)/STBT‐11012(12)/2/2024‐WBSCST SEC) and Investigator‐Initiated Research Proposals ‐SMALL grant from Indian Council of Medical Research (ICMR) (File No. EMDR/SG/14/2025‐00024 dt. 28/01/2026) for providing financial support. IDJ acknowledges ANRF/ SERB for the National Post‐Doctoral Fellowship (PDF/2023/000284).

## Conflicts of Interest

The authors declare no conflicts of interest.

## Supporting information

This file includes Materials, Synthetic procedures, Experimental details, Copies of NMR Spectra, HRMS spectra and HPLC. Supplementary text, methods, and supplementary figures S1‐S20 were also available.  

## Data Availability

The data supporting this article are included in the Supporting Information.
