# Supplementary material for: Rational Design of Glycolipid‐Based Coacervates for Sequestration of Macromolecules and Enveloped Viruses
Source: Small Sci. 2026 Oct 25;6(11):e70399. doi: 10.1002/smsc.70399 (PMC13696755; doi:10.1002/smsc.70399)
Supplement: Supplementary file 1 [file SMSC-6-e70399-s001.pdf]

Rational Design of Glycolipid-based Coacervates for Sequestration of Macromolecules and Enveloped Viruses

Nikesh Dewangan, Sandeep Yadav[#], Sarani Biswas, Indrani Das Jana[§], Ananya Ghosh, Priyaraj Pal, Arindam Mondal[§], Amirul Islam Mallick*[#], and Pradip Kumar Tarafdar*

Department of Chemical Sciences, Indian Institute of Science Education and Research Kolkata, Mohanpur-741246, India

[#]Department of Biological Sciences, Indian Institute of Science Education and Research Kolkata, Mohanpur-741246, India

[§]Department of Bioscience and Biotechnology, Indian Institute of Technology Kharagpur, Kharagpur 721302

*Corresponding authors

This file includes:

Materials

Synthetic procedures

Copies of ^{1}H, HRMS spectra, and HPLC

Supplementary text and methods

Supplementary figures **S1–S20**

References

General Information:

The chemicals, L-amino acids, fluorescence probes, and solvents were purchased from commercial sources (Merck, Alfa Aesar, BIOSYNTH, Sisco Research Laboratories, Avra Synthesis Private Ltd.). Merck precoated silica gel 60 F_{254} aluminium sheets were used for thin-layer chromatography (TLC) analysis. Chromatographic separations were performed on silica gel (60-120 mesh or 100-200 mesh). For rapid purification, flash chromatography was performed on an automated system (Combiflash NEXTGEN 300+) using pre-packed cartridges of silica (50 µm Redisep®Rf Teledyne Isko Column). High-resolution mass spectra (HRMS) were recorded on Bruker Micro-TOF and Waters XEVO G2-XS QTOF spectrometer. NMR spectroscopy of the synthetic compounds was performed on a Bruker AVANCE 500 MHz and a JEOL 400 MHz spectrometer. MestReNova 14.1.2 was used to process all spectra. Milli-Q water was used to prepare the buffer solutions.

Synthesis of glycolipid mimics: -

We synthesised a series of glycolipid mimics, including **MWG, MWGS-A_4, MWGSD-A_4**, and **MGSD-A_4.** These molecules were synthesised using a solution-phase approach described in Figures S1 and S2.

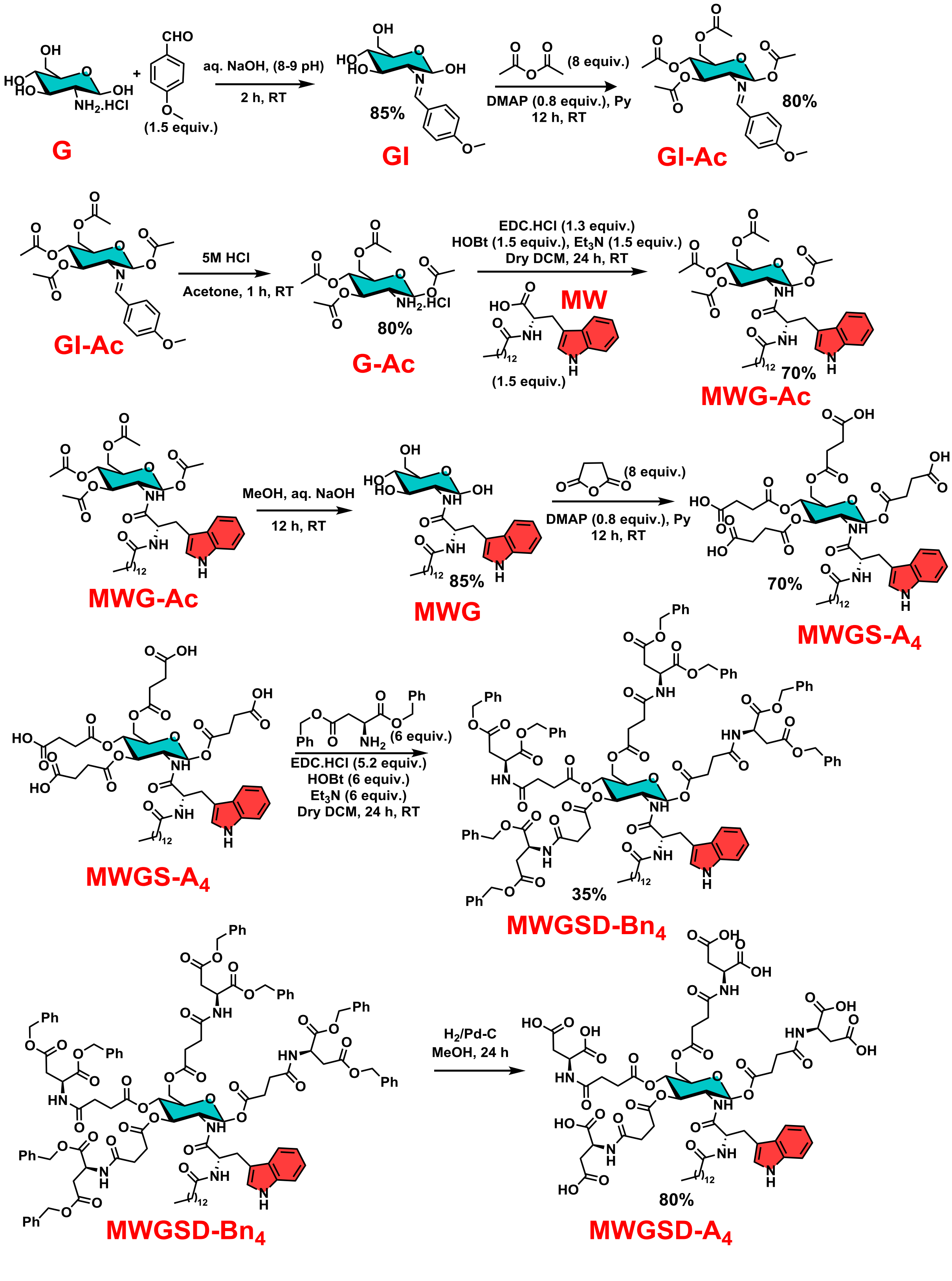

Figure S1: Synthetic scheme of glycolipid mimics with tryptophan.

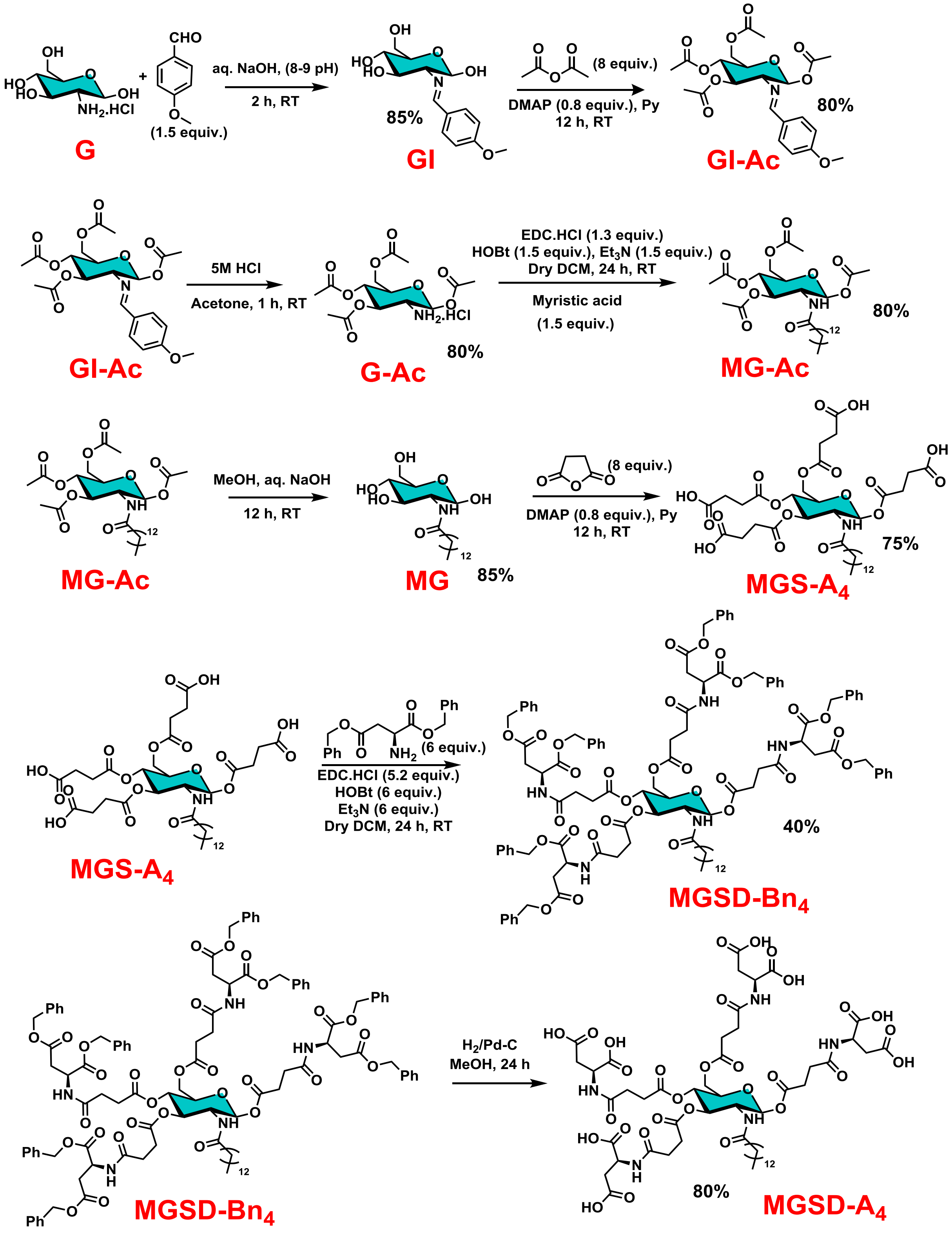

Figure S2: Synthetic scheme for glycolipid mimics lacking tryptophan.

Experimental Procedures:

Glucosamine protection (P1)

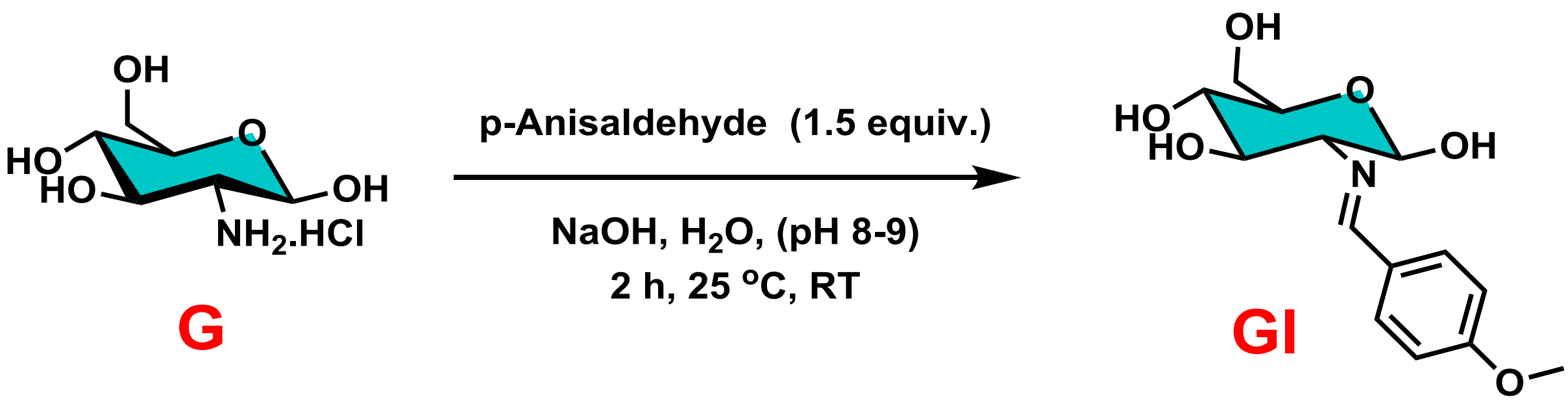

10 g (46.3 mmol) of glucosamine hydrochloride (G) was dissolved in 20 mL of water. Aqueous NaOH was added dropwise to maintain a pH of 8-9. The reaction mixture was then cooled in an ice bath for 10 minutes. Subsequently, *p*-anisaldehyde (8.46 mL, 69.56 mmol) was added gradually, and the reaction mixture was stirred at room temperature for 2 hours. The resulting white solid was collected by filtration using Whatman filter paper and washed with diethyl ether. The obtained solid (glucosamine imine, GI) was dried and characterised by ^{1}H NMR spectroscopy.

Synthesis of acetylated glucosamine imine (GI-Ac) (P2)

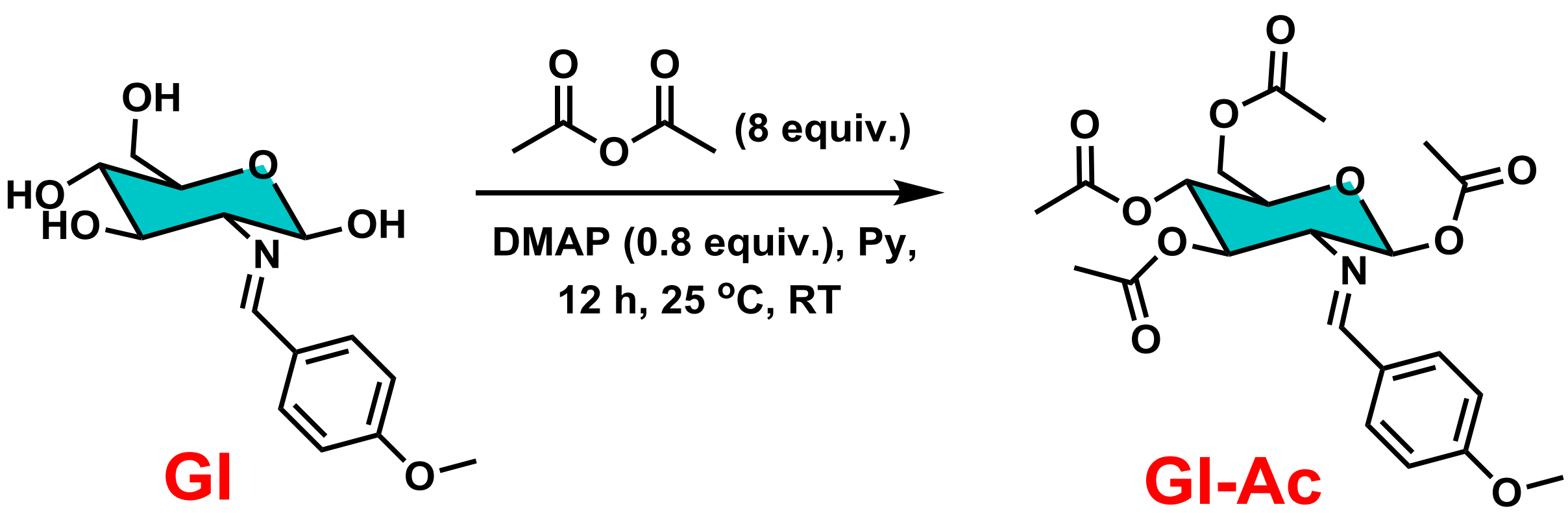

Glucosamine imine (10 g, 33.67 mmol) and DMAP (3.29 g, 26.93 mmol) were dissolved in 25 mL of pyridine (Py) and kept on ice for 10-15 minutes. Acetic anhydride (25.42 ml, 269 mmol) was added slowly, and the mixture was stirred overnight at room temperature. Crushed ice was added to the reaction mixture, yielding a white precipitate. The solid was

filtered using Whatman filter paper and dried using a rotary evaporator. The product (acetylated glucosamine imine) was characterised by ^{1}H NMR spectroscopy.

Deprotection of acetylated glucosamine imine (G-Ac) (P3)

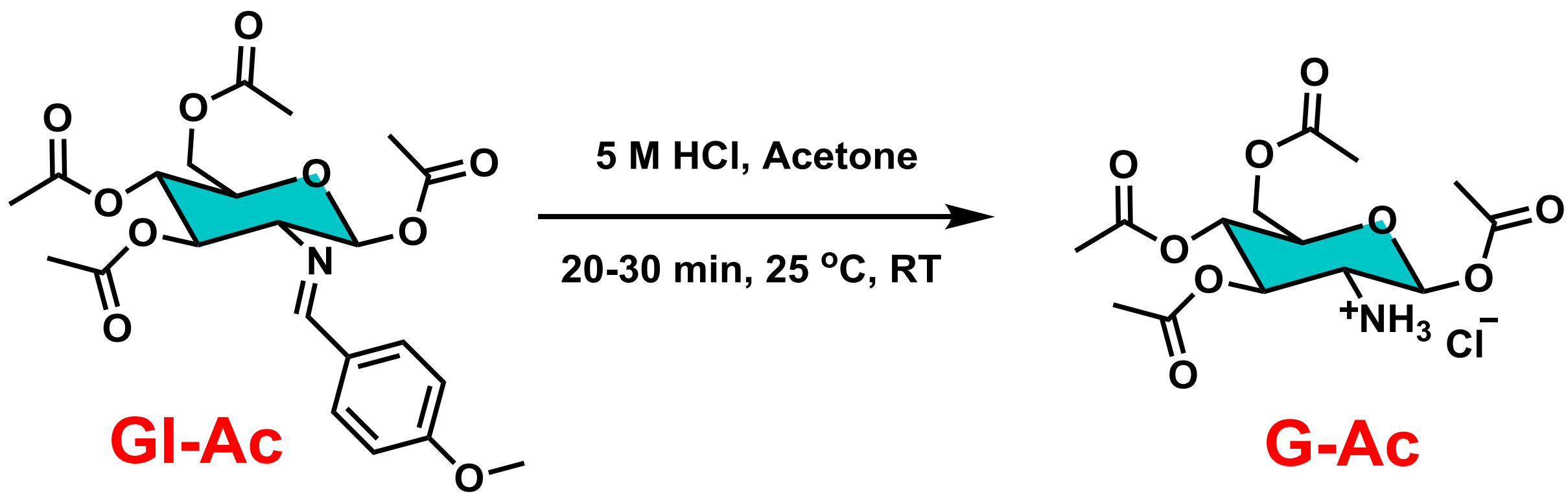

Acetylated glucosamine imine (5 g) was dissolved in 30 mL of acetone in a 100 mL round-bottom flask. 10 mL 5M HCl was added, and the reaction mixture was stirred for 20-30 minutes. The white solid (acetylated glucosamine) was formed during scratching. The solid was filtered using Whatman filter paper, washed with acetone, and dried. The product (acetylated glucosamine) was characterised by ^{1}H NMR spectroscopy.

Synthesis of MWG-Ac (P4)

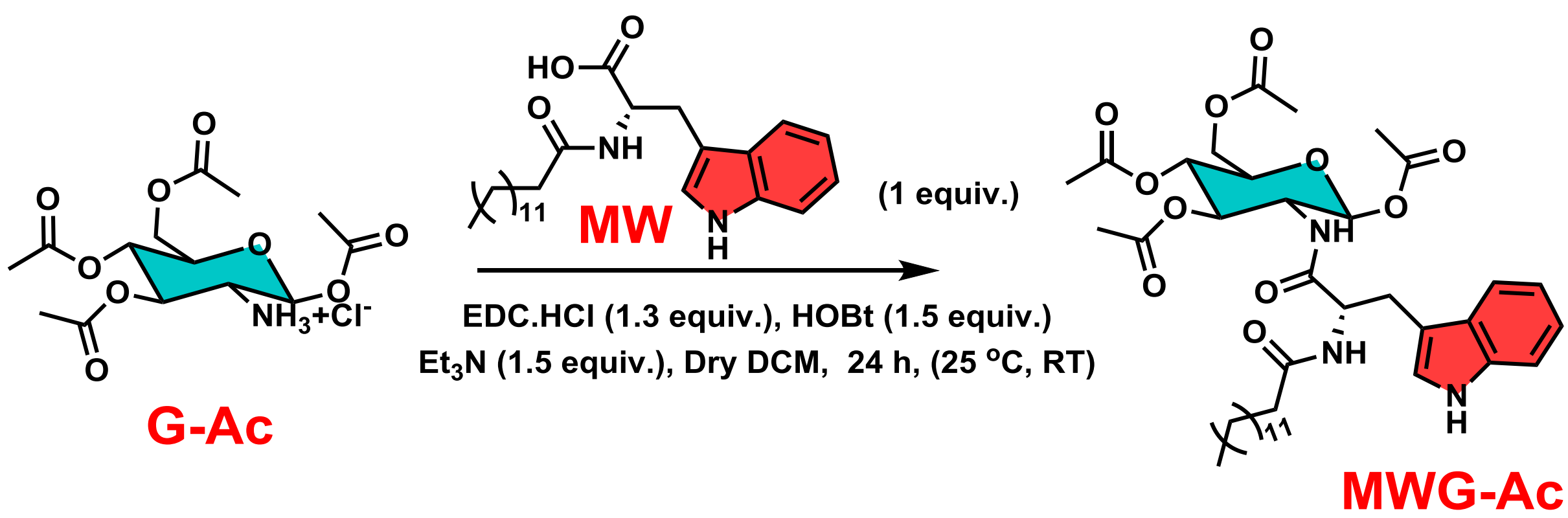

Myristoylated tryptophan (MW) was synthesised by P14-16. 3 g MW (7.24 mmol) was dissolved in 20 mL of dry DCM at an ice-water bath. Acylated glucosamine hydrochloride (4.16 g, 10.86 mmol), EDC·HCl (1.8 g, 9.4 mmol), HOBt (1.46 g, 10.86 mmol), and Et_3N (1.5 mL, 10.86 mmol) were added to the DCM. The reaction was carried out under nitrogen at room temperature for 24 hours with stirring. After the reaction was complete, the organic

layer was washed with 2 M HCl, brine, 1 M sodium carbonate, and brine again. The organic layer was dried with anhydrous sodium sulphate, and the solvent was evaporated under vacuum. The product was purified using silica gel (100-200 mesh) and characterised by ^{1}H NMR spectroscopy.

Synthesis of myristoylated tryptophan glucosamine (MWG) (P5)

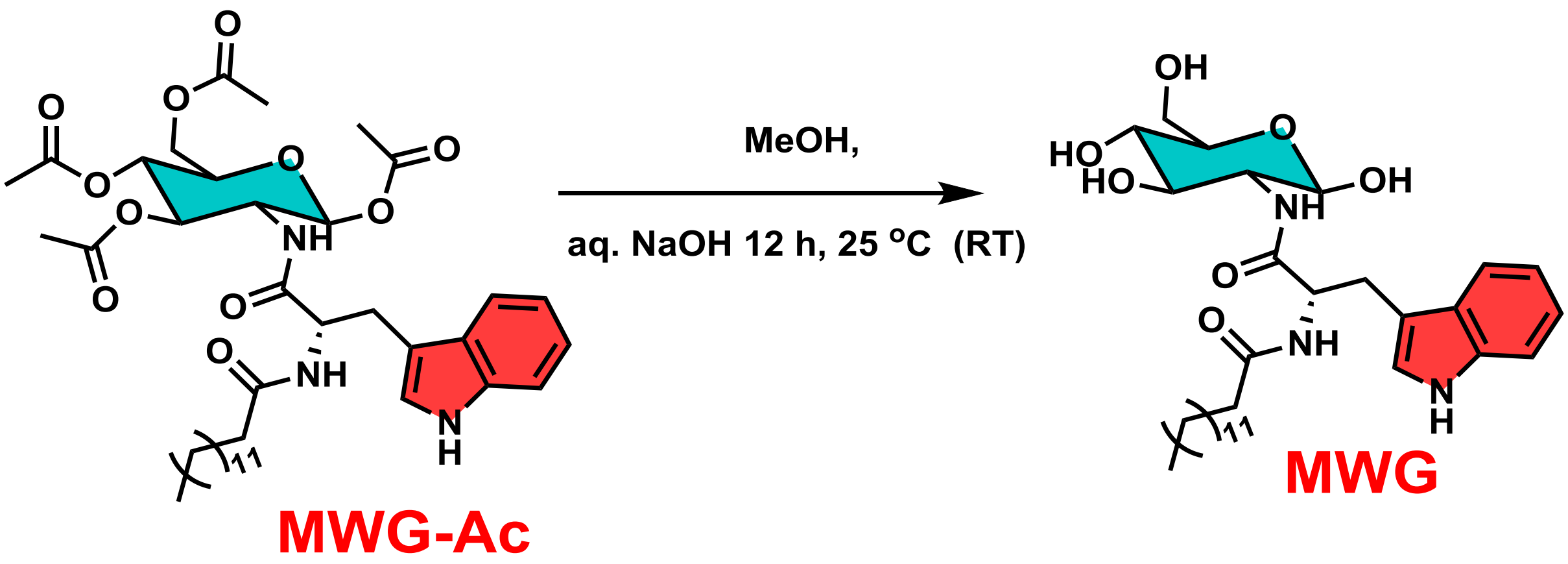

MWG-Ac (2 g) was dissolved in methanol and taken in a round-bottom flask. Then aqueous NaOH was added, and the reaction was stirred at room temperature for 12 hours. After that, the reaction mixture was concentrated by evaporating methanol and acidified with dilute HCl. The precipitate (MWG) was filtered, washed with ethyl acetate and water. MWG was characterised using ^{1}H NMR spectroscopy and HRMS.

Synthesis of myristoylated tryptophan glucosamine succinic acid (MWGS-A$_4$) (P6)

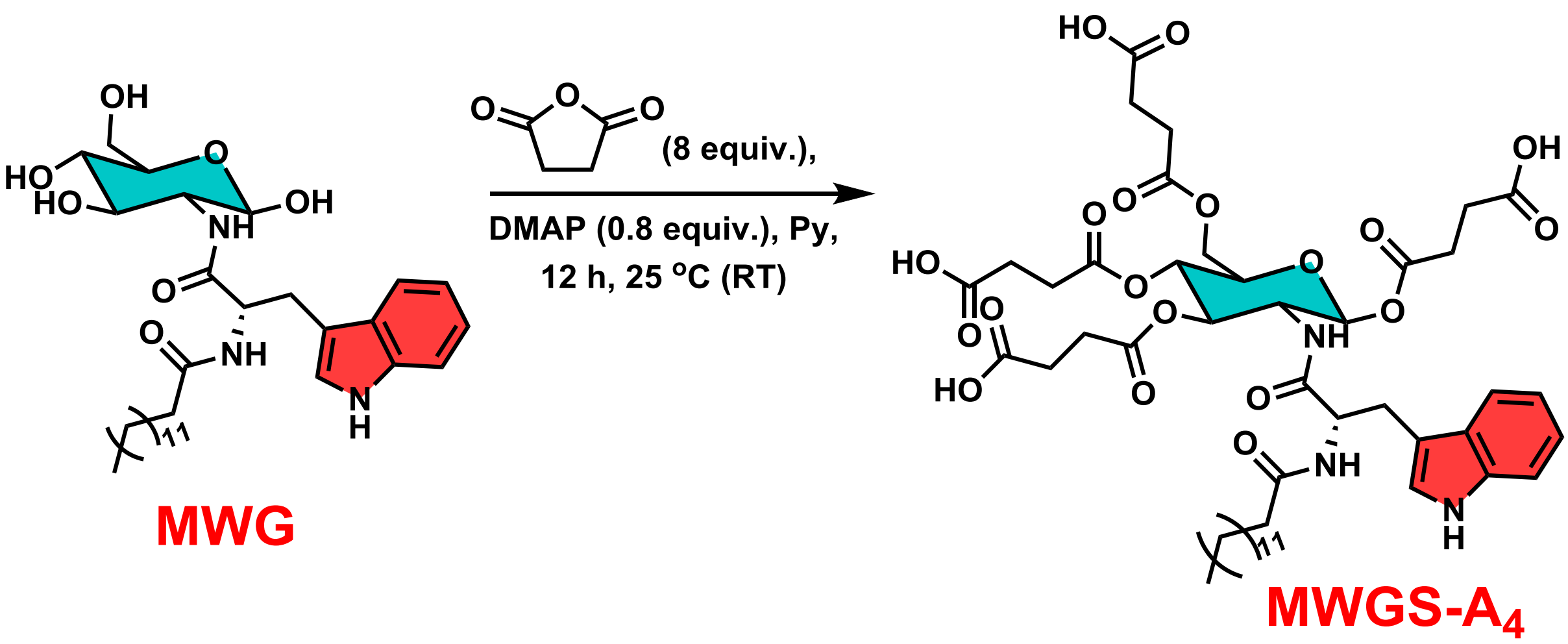

MWG (750 mg, 1.30 mmol) and DMAP (127 mg, 1.04 mmol) were dissolved in 25 mL of pyridine and taken in a 100 mL round-bottom flask. The flask was placed in a cold bath and kept for 10-15 minutes. 1.04g (10.42 mmol) of succinic anhydride (dissolved in DCM and acetone) was added slowly to the pyridine solution. The reaction mixture was stirred for 12 hours at room temperature. Once the reaction was complete, the reaction mixture was concentrated using a rotavapor. The reaction mixture was acidified with dilute HCl, and the organic layer was washed three times with DCM and water. The product was dried, washed with diethyl ether to purify it, and dried again. The product (MWGS-A_4) was characterised by ^{1}H NMR spectroscopy and HRMS.

Synthesis of MWGSD-Bn_4 (P7)

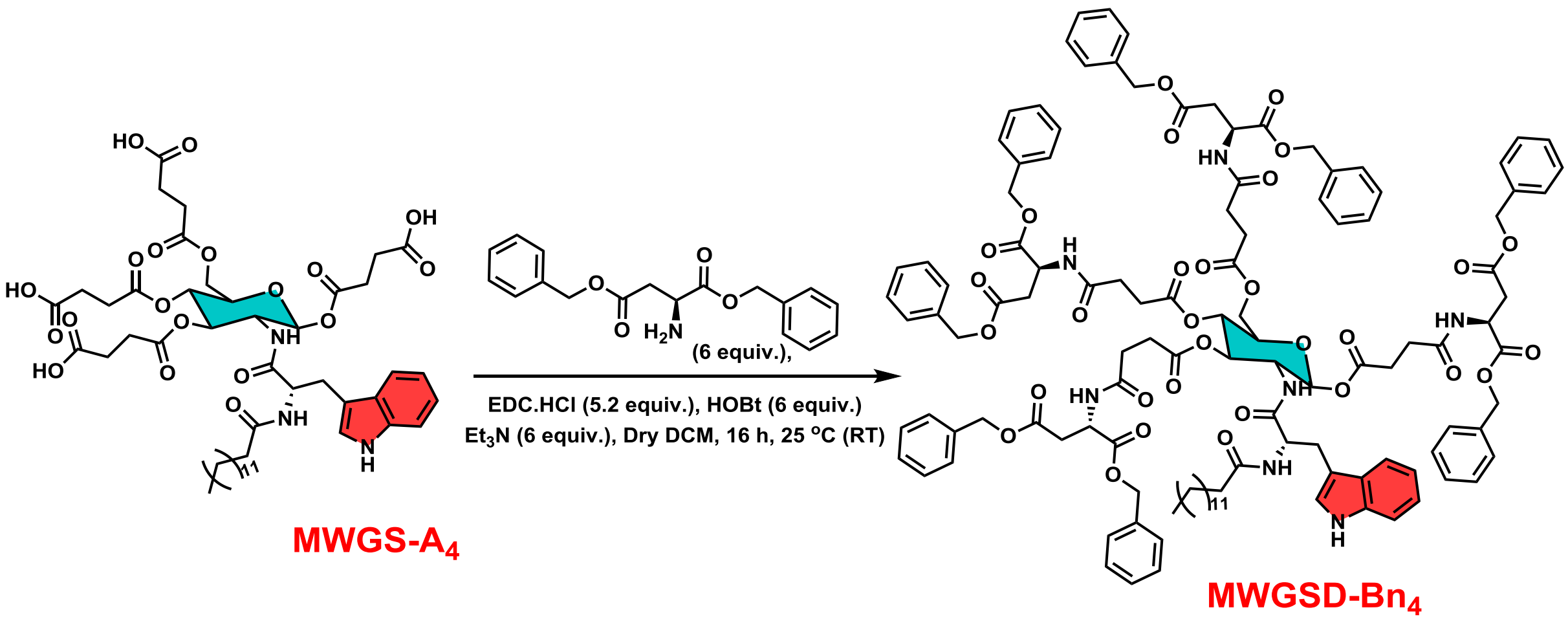

MWGS-A_4 (500 mg, 0.51 mmol) was dissolved in 20 mL of dry DCM and placed at an ice-water bath. Et_3N (0.42 mL, 3.07 mmol), EDC.HCl (509 mg, 2.66 mmol), dibenzyl aspartic acid (961 mg, 3.07 mmol), and HOBt (414 mg, 3.07 mmol) were added, and the reaction was stirred in a nitrogen environment for 16 hours at room temperature. After the reaction was complete, the organic layer (DCM) was washed with 2 M HCl, brine, and 1 M sodium carbonate. The organic layer was concentrated and dried with anhydrous sodium sulphate. MWGSD-Bn_4 was purified with silica gel (200-400 mesh) using DCM: methanol as eluting solvent under flash chromatography. The product (MWGSD-Bn_4) was characterised by ^{1}H NMR spectroscopy and HRMS.

Synthesis of myristoylated tryptophan glucosamine succinate tetra aspartic acid (MWGSD-A_4) (P8)

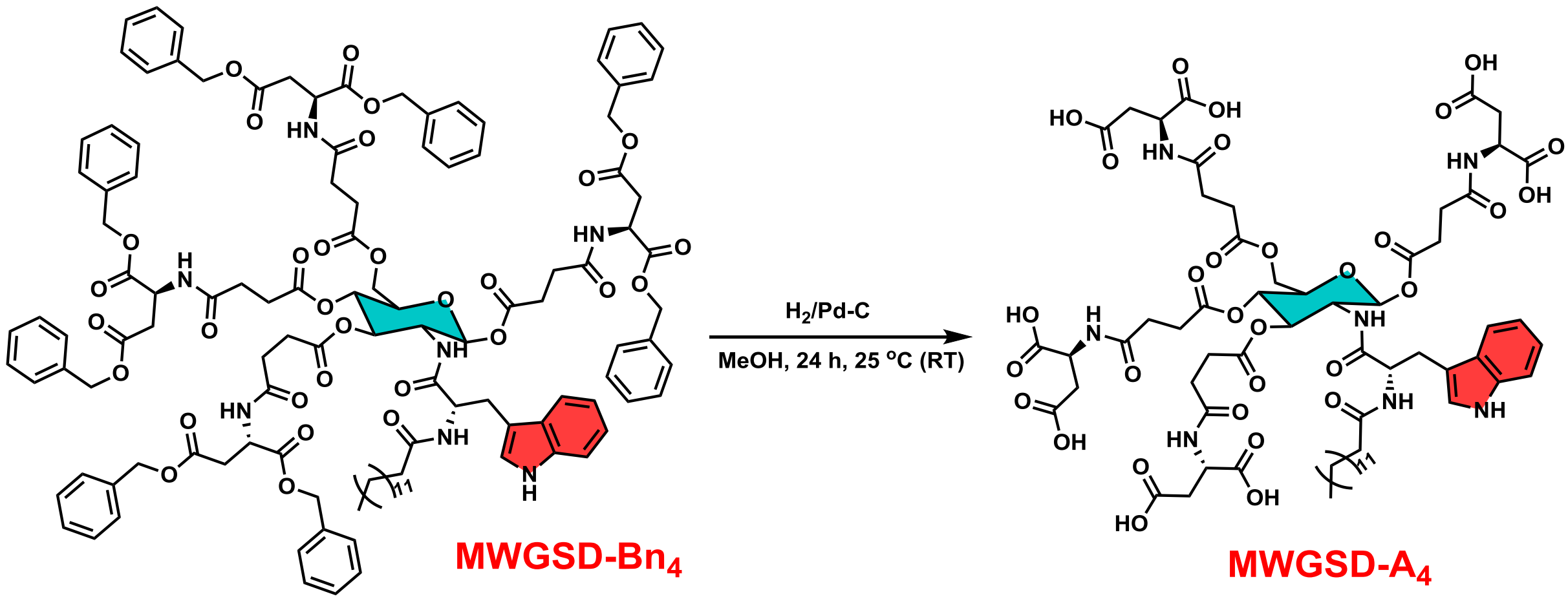

MWGSD-Bn$_4$ (200 mg) was taken in a 100 mL RB flask, and 20 mL of methanol was added to dissolve the compound. Next, 100 mg 10% Pd/C was added, and the reaction mixture was agitated for 30 to 60 minutes while purging with N_2. The H_2 balloon was attached to the reaction mixture, and agitation was continued for 24 hours at room temperature (25 °C). The TLC was monitored to check the completion of the reaction. The Pd/C was filtered through Celite using a sintered funnel, and the product was obtained by evaporating the methanol. The product (MWGSD-A$_4$) was analysed by using ^{1}H NMR spectroscopy, HRMS and HPLC.

Synthesis of myristoylated glucosamine-Ac (MG-Ac) (P9)

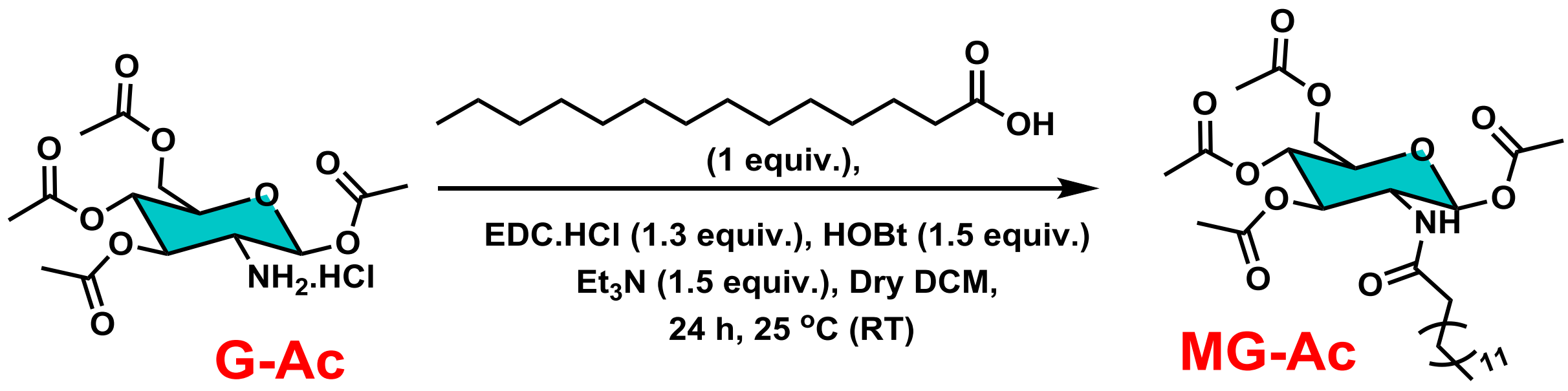

Myristic acid (1 g, 4.38 mmol) was dissolved in 20 mL of dry DCM and placed in an ice-water bath. Acylated glucosamine hydrochloride (2.52 g, 6.57 mmol), EDC·HCl (1.09 g, 5.694 mmol), HOBt (887 mg, 6.57 mmol), and Et_3N (0.9 ml, 6.57 mmol) were added to DCM. The reaction was carried out under nitrogen at room temperature for 24 hours with stirring. After the reaction, the organic layer was washed with 2 M HCl, brine, 1 M sodium carbonate, and brine again. The organic layer was dried with anhydrous sodium sulphate, and

concentrated under vacuum. The product was purified using silica gel (100-200 mesh) and characterised by ^{1}H NMR spectroscopy.

Synthesis of myristoylated glucosamine (MG) (P10)

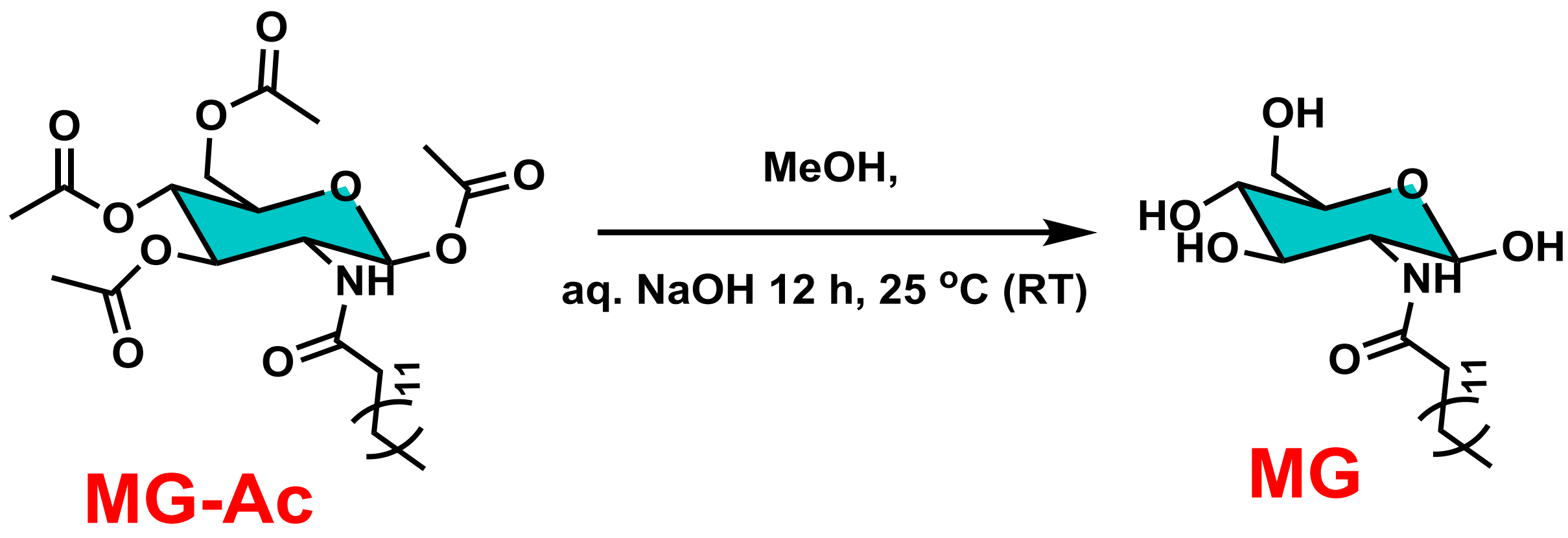

MG-Ac (2 g) was taken in an RB flask and dissolved in methanol. Aqueous NaOH was added, and the reaction mixture was stirred at room temperature for 12 hours. The methanol was evaporated in a rotavapor, and the solution was acidified with dilute HCl. The product (MG) was extracted with ethyl acetate. Next, the ethyl acetate extract was dried over Na_2SO_4 and evaporated to yield a solid product of Myr-glucosamine (MG). MG was analysed using ^{1}H NMR spectroscopy.

Synthesis of myristoylated glucosamine succinic acid (MGS-A$_4$) (P11)

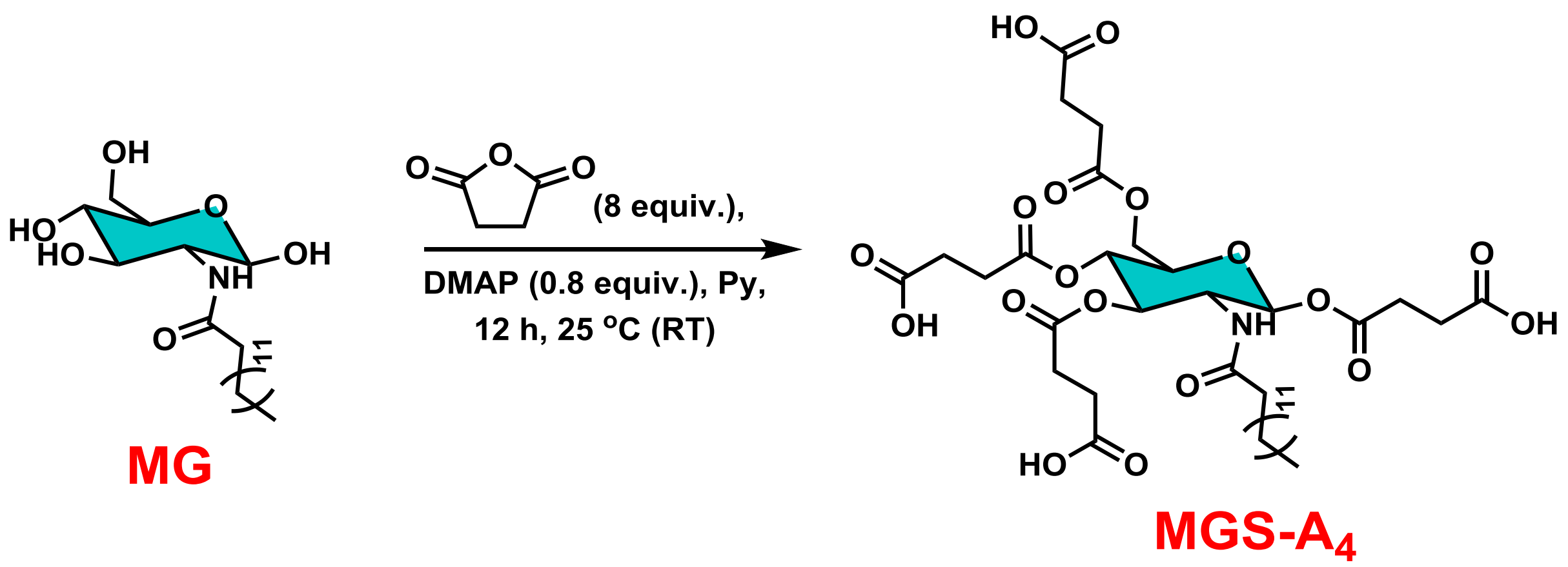

MG (1 g, 2.56 mmol) and DMAP (0.25 g, 2.05 mmol) were dissolved in 25 mL of pyridine and taken in a 100 mL round-bottom flask. The flask was placed in a cold bath for 10-15 minutes. Next, 2.05 g (20.53 mmol) of succinic anhydride (dissolved in DCM and acetone) was added

to the pyridine solution. The reaction mixture was stirred for 12 hours at room temperature. Once the reaction was complete, the reaction mixture was concentrated using a rotavapor. The reaction mixture was acidified with dilute HCl, and the organic layer was washed three times with DCM and water. The product was dried, washed with diethyl ether to purify it, and dried again. The product (MGS-A_4) was characterised using ^{1}H NMR spectroscopy.

Synthesis of MGSD-Bn_4 (P12)

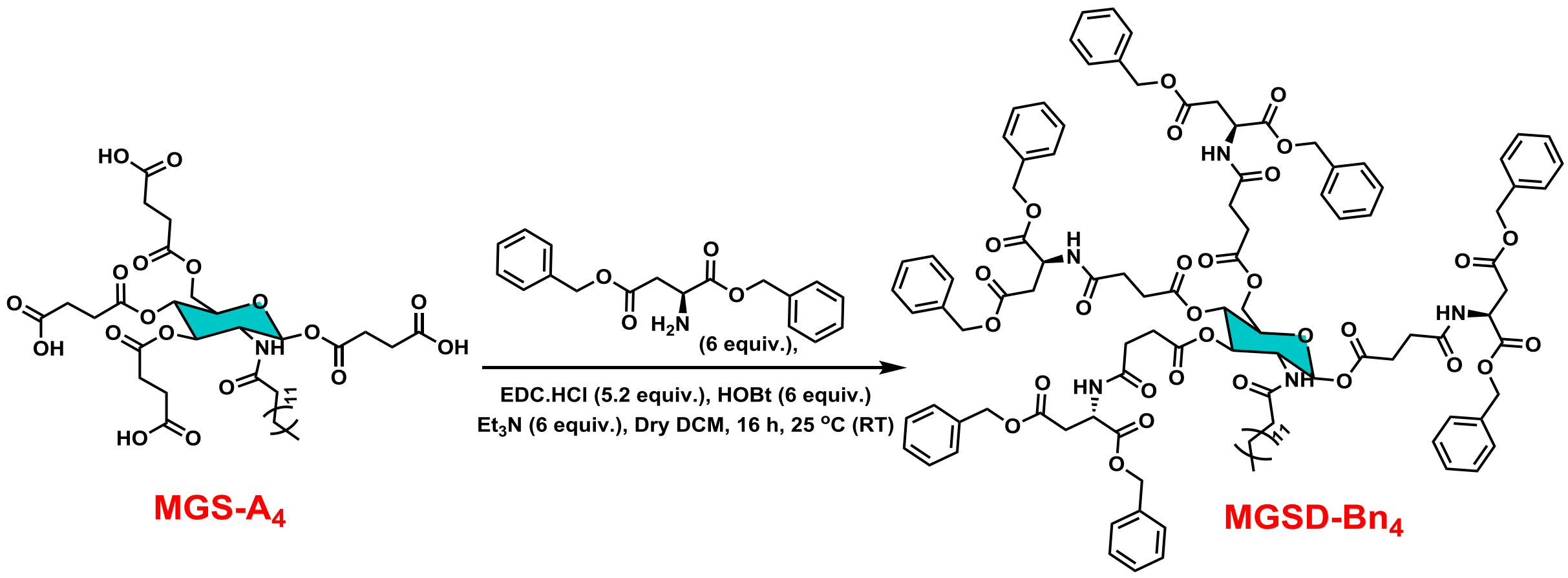

MGS-A_4 (1 g, 1.266 mmol) was dissolved in 20 mL of dry DCM and placed at an ice-water bath. Et_3N (1.05 ml, 7.59 mmol), EDC.HCl (1.258 g, 6.58 mmol), dibenzyl aspartic acid (2.378 g, 7.59 mmol), and HOBt (1.025 g, 7.59 mmol) were added, and the reaction was stirred in a nitrogen environment for 16 hours at room temperature. After the reaction was completed, the organic layer (DCM) was washed with 2 M HCl, brine, and 1 M sodium carbonate. The organic layer was concentrated and dried with anhydrous sodium sulphate. MGSD-Bn_4 was purified with silica gel (200-400 mesh) using DCM: methanol as eluting solvent under flash chromatography. The product (MGSD-Bn_4) was characterised by ^{1}H NMR spectroscopy and HRMS.

Synthesis of myristoylated glucosamine succinate tetra aspartic acid (MGSD-A_4) (P13)

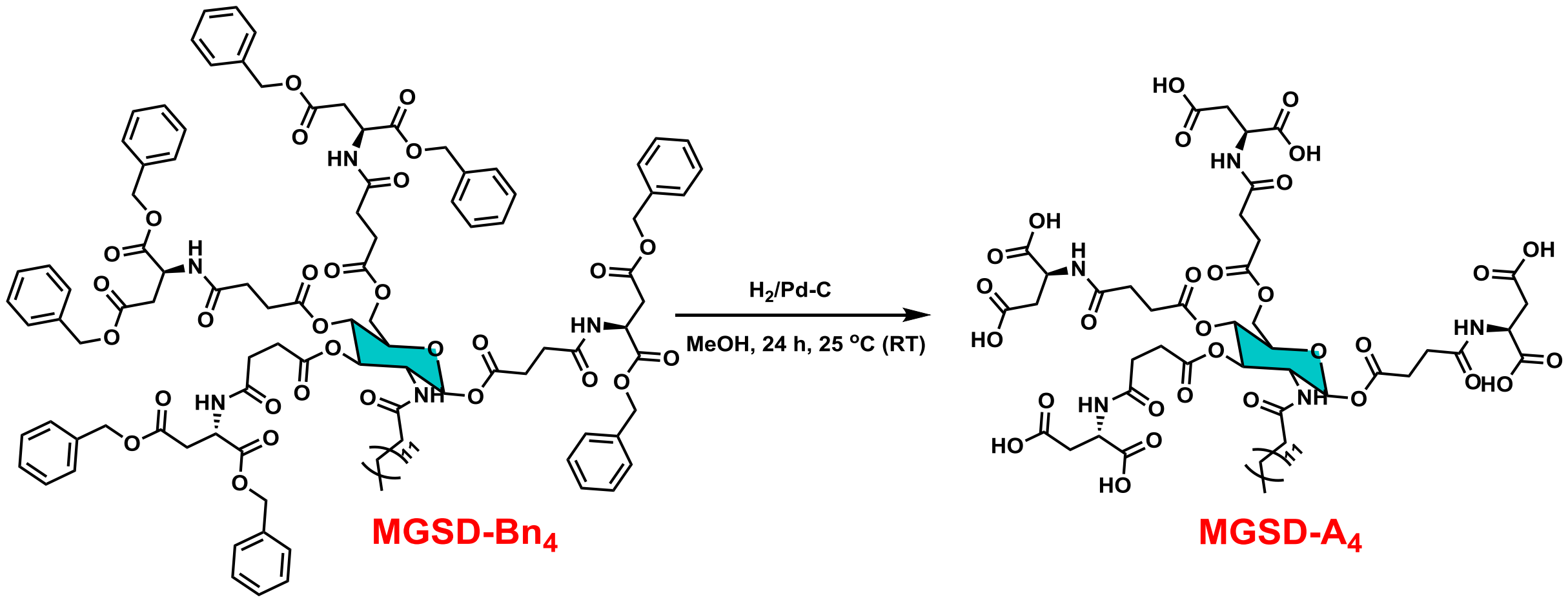

MGSD-Bn$_4$ (200 mg) was taken in a 100 mL RB flask, and 20 mL of methanol was added to dissolve the compound. Next, 100 mg 10% Pd/C was added, and the reaction mixture was agitated for 30 to 60 minutes while purging with N_2. The H_2 balloon was attached to the reaction mixture, and agitation was continued for 24 hours at room temperature (25 °C). The TLC was monitored to check the completion of the reaction. The Pd/C was filtered through Celite, and the product was obtained by evaporating the methanol. The product (MGSD-A$_4$) was analysed by using ^{1}H NMR spectroscopy.

Synthesis of tryptophan methyl ester (WE) (P14)

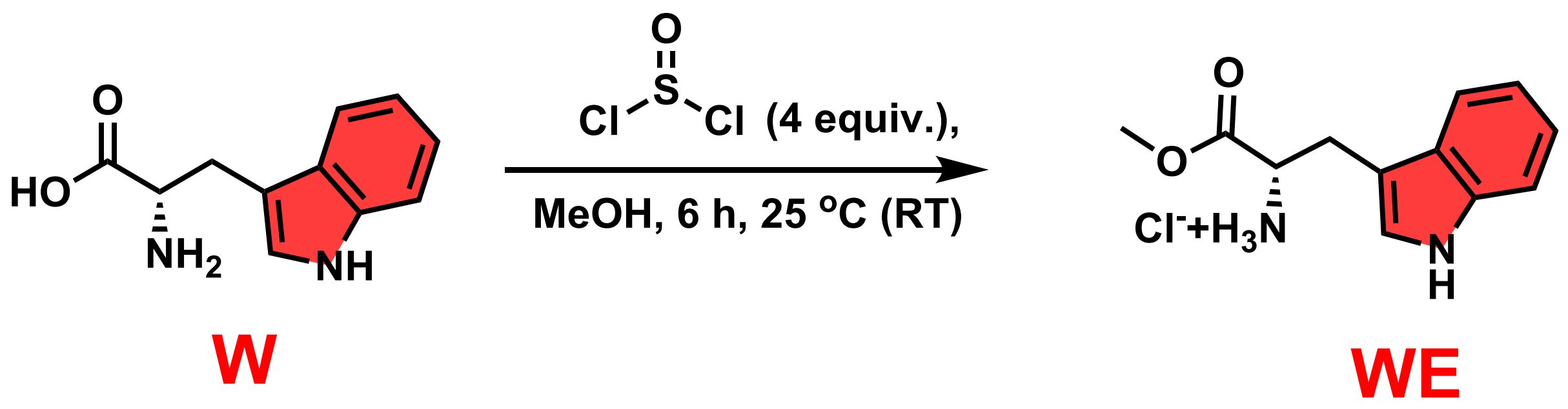

L-Tryptophan (5 g, 24.5 mmol) was dissolved in dry methanol at 0 °C. Then thionyl chloride (7.1 mL, 98.0 mmol) was added dropwise, and the reaction mixture was stirred for six hours. After the reaction was complete, the excess thionyl chloride was removed by purging with nitrogen (N_2). The concentrated product was then washed with hexane. Finally, the methyl ester of tryptophan was obtained.

Synthesis of myristoylated tryptophan methyl ester (MWE) (P15)

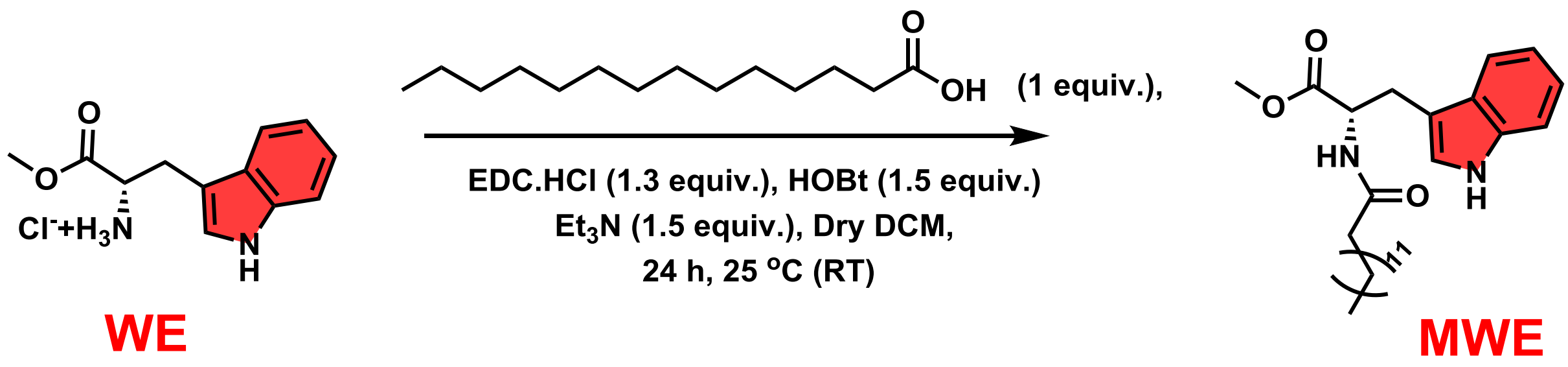

Myristic acid (1 g, 4.37 mmol) was dissolved in 20 mL of dry DCM and placed in an ice-water bath. Methyl ester of tryptophan hydrochloride (1.670 g, 6.56 mmol), EDC·HCl (1.09 g, 5.694 mmol), (1.091 g, 5.69 mmol), HOBt (0.886 g, 6.56 mmol), and Et_3N (0.91 ml, 6.56 mmol), were added to DCM. The reaction was carried out under nitrogen at room temperature for 24 hours with stirring. After the reaction, the organic layer was washed with 2 M HCl, brine, 1 M sodium carbonate, and brine again. The organic layer was dried with anhydrous sodium sulphate, and concentrated under vacuum. The product was purified using silica gel (100-200 mesh) and characterised by ^{1}H NMR spectroscopy.

Synthesis of myristoylated tryptophan (MW) (P16)

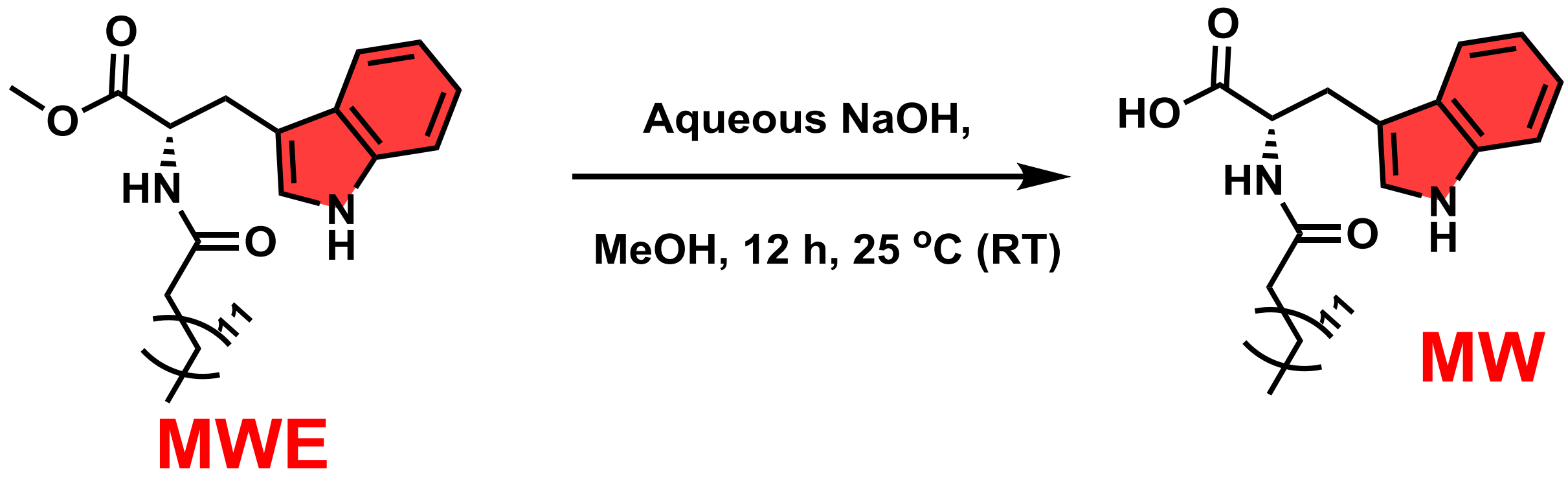

MWE (4 g) was taken in an RB flask and dissolved in methanol. Aqueous NaOH was added, and the reaction mixture was stirred at room temperature for 12 hours. The methanol was evaporated in a rotavapor, and the solution was acidified with dilute HCl. The product (MW) was extracted with ethyl acetate. Next, the ethyl acetate extract was dried over Na_2SO_4 and evaporated to yield a solid product of Myr-tryptophan (MW). MW was analysed using ^{1}H NMR spectroscopy.

Synthetic steps and characterisation of the compounds:

Synthesis of MWG: P1 (Yield: 85%), P2 (Yield: 80%), P3 (Yield: 80%), P4 (Yield: 70%) and P5 (85%). ^{1}H NMR spectrum of MWG (500 MHz, DMSO-D_6).

^{1}H NMR (500 MHz, DMSO) δ 10.76 (s, 1H), 7.86 (dd, *J* = 8.4 Hz, 1H), 7.74 (d, *J* = 6.1 Hz, 1H), 7.69 – 7.58 (dd, , *J* = 7.9 Hz, 1H), 7.30 (dd, *J* = 8.0, 4.6 Hz, 1H), 7.13 (d, *J* = 17.5 Hz, 1H), 7.04 (dd, *J* = 7.9 Hz, 1H), 6.95 (dd, *J* = 7.3, 7.2 Hz, 1H), 4.96 (d, *J* = 7.1 Hz, 1H), 4.72 – 4.45 (m, 2H), 3.70 – 3.55 (m, 4H), 3.54 – 3.32 (m, 2H), 3.20 – 3.09 (m, 4H), 2.90 (t, *J* = 12.3 Hz, 1H), 2.06 – 1.96 (t, 2H), 1.40 – 1.30 (m, 2H), 1.26 – 0.99 (m, 20H), 0.86 (t, *J* = 6.7 Hz, 3H).

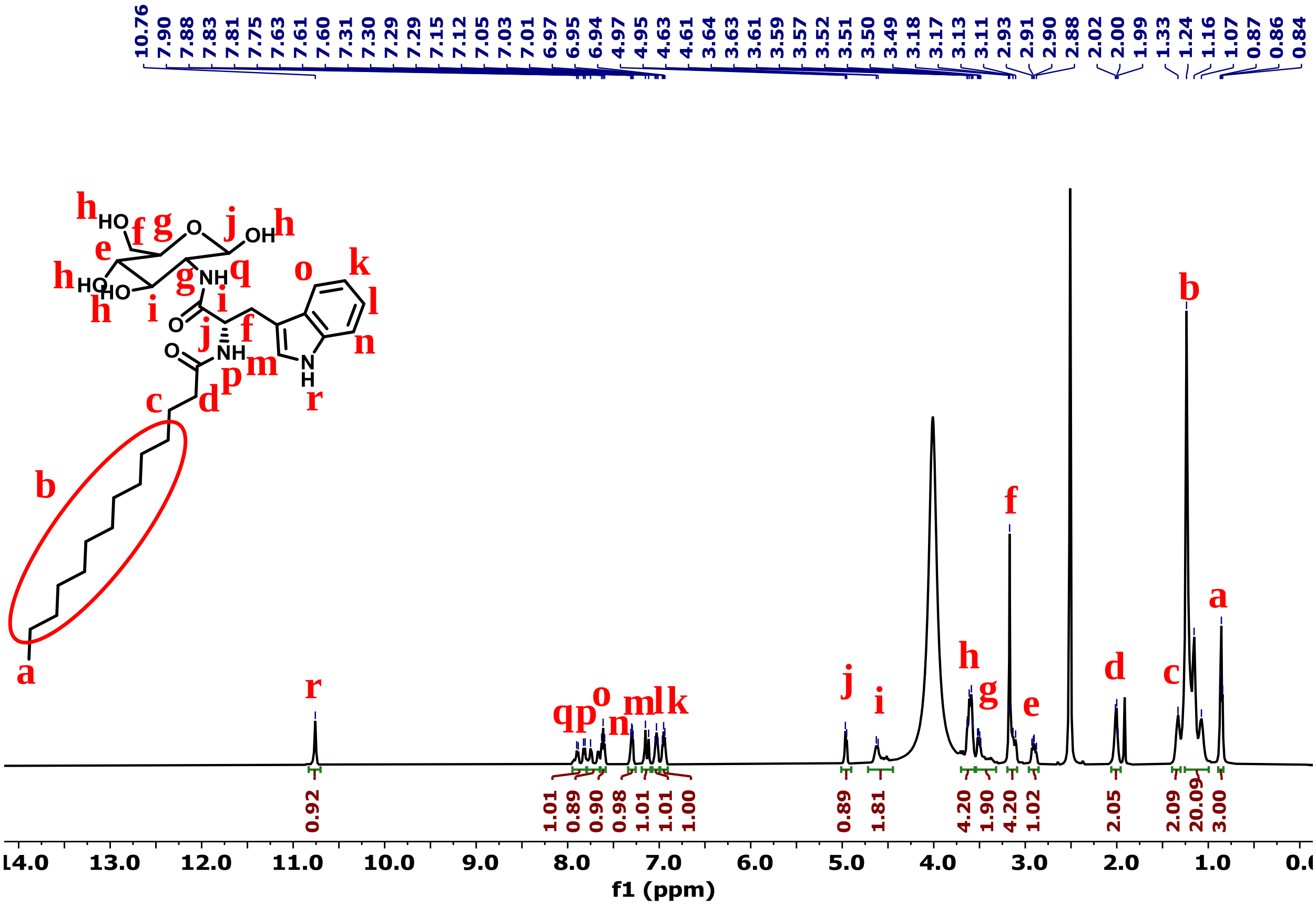

Figure S3: ^{1}H NMR of MWG.

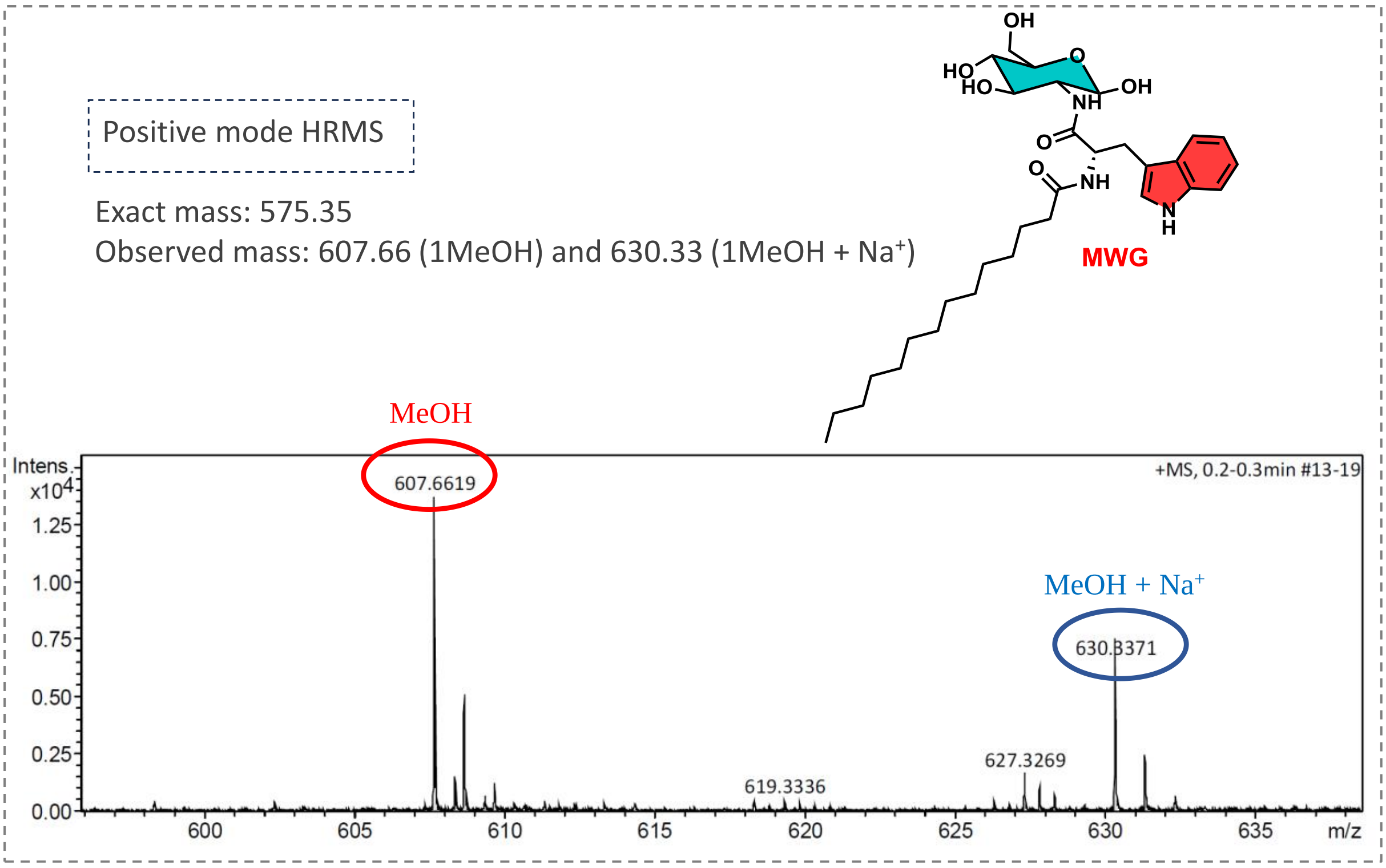

Figure S4: HRMS of MWG.

Synthesis of MWGS-A$_4$: P1 (Yield: 85%), P2 (Yield: 80%), P3 (Yield: 80%), P4 (Yield: 70%), P5 (Yield: 85%), and P6 (Yield: 70%). ^{1}H NMR spectra of myristoylated tryptophan glucosamine succinic acid (MWGS-A$_4$): (500 MHz, DMSO-D_6).

^{1}H NMR (500 MHz, DMSO) δ 12.22 (s, 4H), 10.75 (s, 1H), 8.04 (d, *J* = 8.4 Hz, 1H), 7.91 (t, *J* = 6.9 Hz, 1H), 7.57 (dd, *J* = 8.2 Hz, 1H), 7.31 (dd, *J* = 8.0, 4.6 Hz, 1H), 7.11 (s, 1H), 7.06 (dd, *J* = 7.9 Hz, 1H), 6.98 (dd, *J* = 7.3, 7.2 Hz, 1H), 5.81 (m, 1H), 5.32 (m, 1H), 4.96 (dd, *J* = 9.7, 4.5 Hz, 1H), 4.30 (m, 1H), 4.16 (m, 1H), 4.06 (dd, *J* = 26.6, 13.2 Hz, 3H), 2.97 (m, 1H), 2.91 (m, 2H), 2.83 (m, 1H), 2.58 (m, 2H), 2.50 – 2.40 (m, 8H), 2.41 (m, 2H), 2.38 (m, *J* = 3.6 Hz, 2H), 2.00 (t, *J* = 6.4 Hz, 2H), 1.34 (m, 2H), 1.20 (m, 20H), 0.86 (t, *J* = 6.7 Hz, 3H).

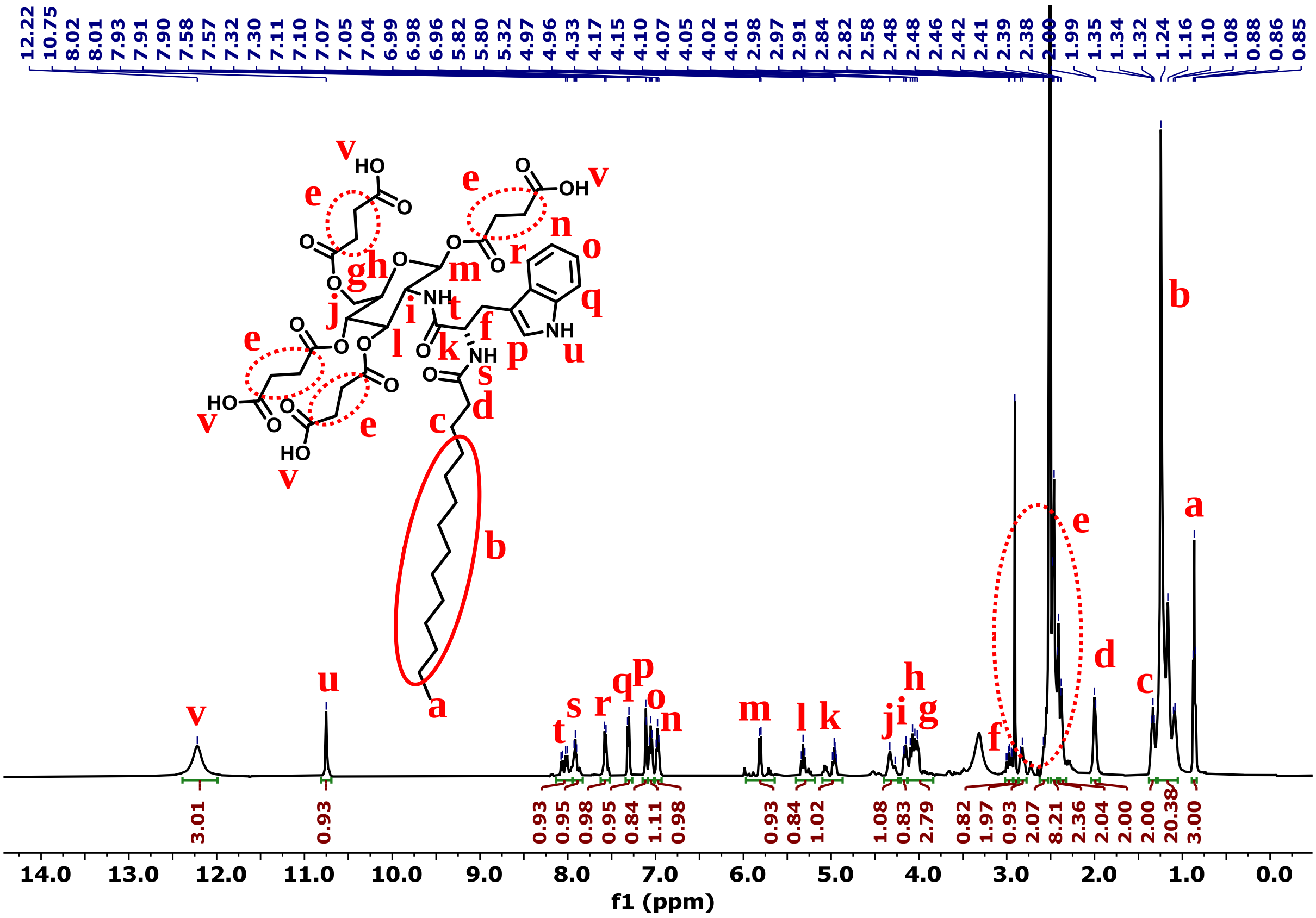

Figure S5: ^{1}H NMR of myr-tryptophan glucosamine tetra succinic acid (MWGS-A_4).

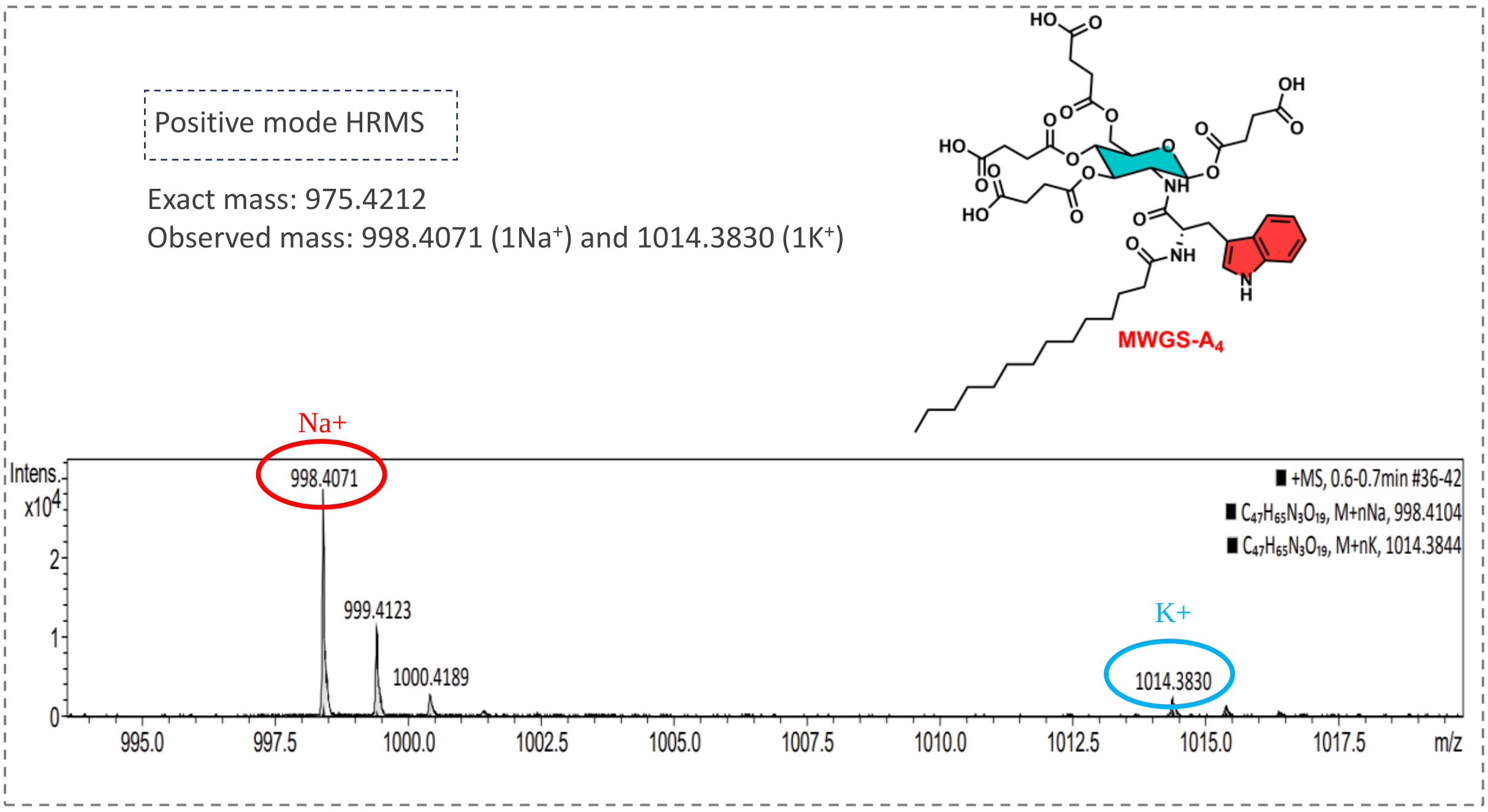

Figure S6: HRMS of MWGS-A_4.

Synthesis of myr-tryptophan glucosamine succinate benzyl protected aspartic acid (MWGSD-(Bn)$_4$): P1 (Yield: 85%), P2 (Yield: 80%), P3 (Yield: 80%), P4 (Yield: 70%), P5 (Yield: 85%), P6 (Yield: 70%), P7 (35%) ^{1}H NMR spectra of MWGSD-Bn$_4$: (500 MHz, DMSO-D_6).

^{1}H NMR (500 MHz, $CDCl_3$) δ 9.31 – 8.79 (m, 1H), 7.62 (q, J = 6.7 Hz, 1H), 7.37 – 7.29 (m, 32H), 7.28 – 7.24 (m, 8H), 7.21 – 7.16 (m, 1H), 7.16 – 7.12 (m, 1H), 7.12 – 7.09 (m, 1H), 7.09 – 7.04 (m, 1H), 7.01 – 6.81 (m, 3H), 6.75 (dd, J = 8.2, 8.2 Hz, 1H), 6.36 – 6.05 (m, 2H), 5.16 – 5.02 (m, 16H), 4.98 – 4.88 (m, 4H), 4.85 – 4.68 (m, 1H), 4.47 – 4.34 (m, 1H), 4.27 – 4.16 (m, 1H), 4.09 – 3.93 (m, 2H), 3.91 – 3.74 (m, 1H), 3.28 – 3.18 (m, 2H), 3.13 – 2.99 (m, 4H), 2.97 – 2.84 (m, 4H), 2.70 – 2.58 (m, 4H), 2.58 – 2.31 (m, 12H), 2.18 – 2.07 (m, 2H), 1.59 – 1.49 (m, 2H), 1.32 – 1.24 (m, 20H), 0.90 (t, J = 6.9 Hz, 3H).

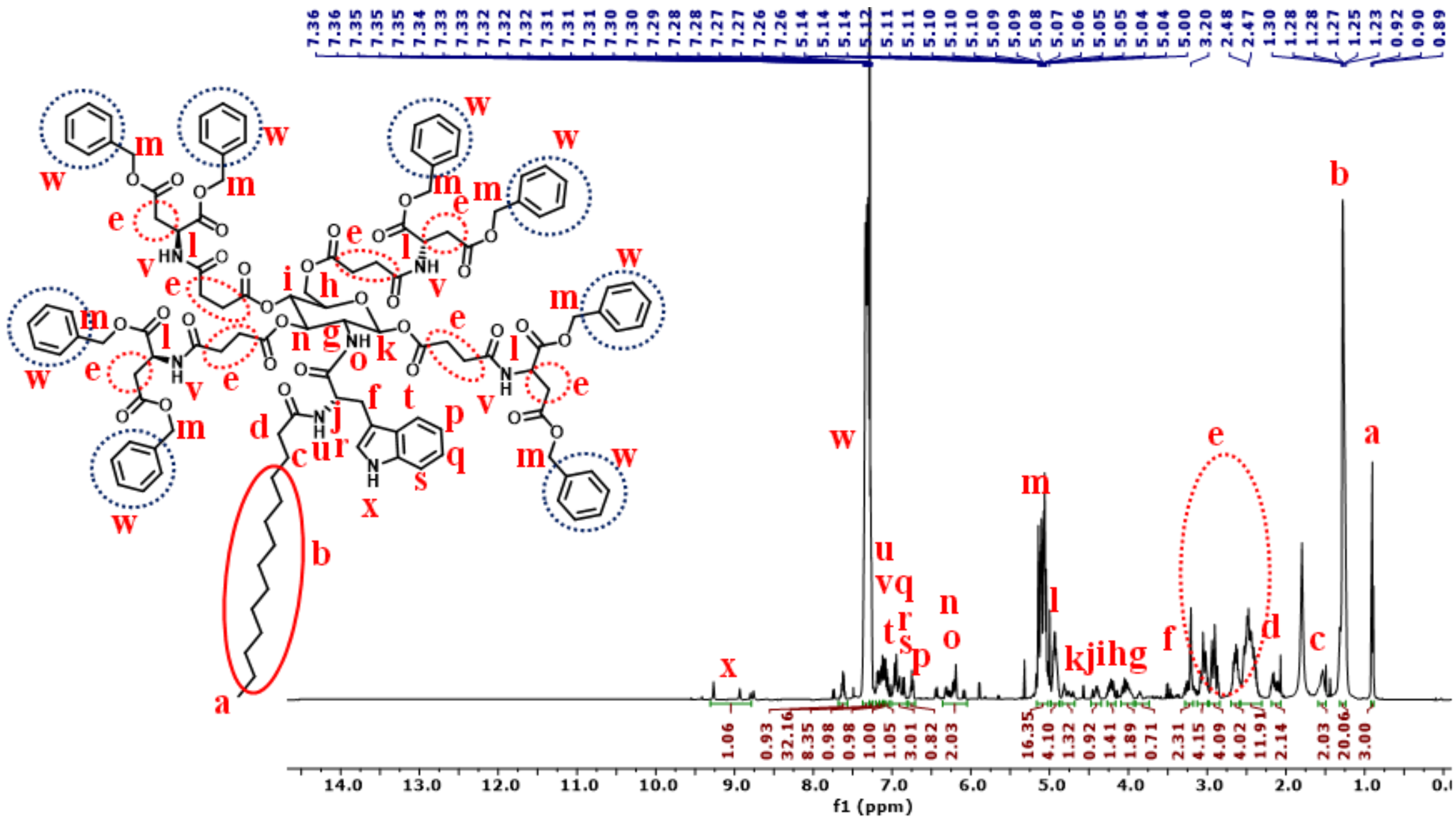

Figure S7: ^{1}H NMR of myr-tryptophan glucosamine tetra succinate tetra benzyl aspartate (MWGSD-Bn$_4$).

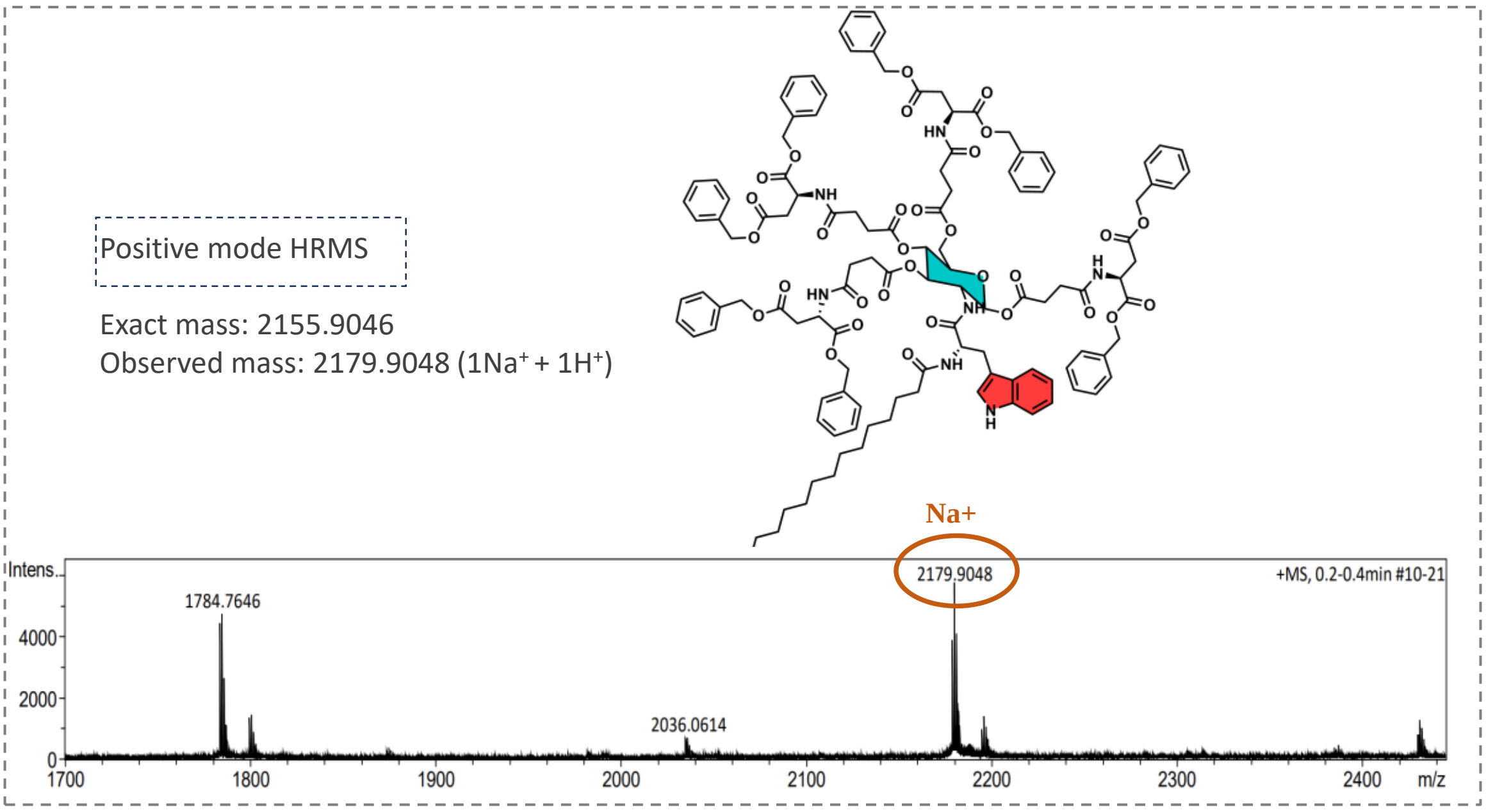

Figure S8: HRMS of MWGSD-Bn_4.

Synthesis of myr-tryptophan glucosamine succinate tetra aspartic acid (MWGSD-A_4): P1 (Yield: 85%), P2 (Yield: 80%), P3 (Yield: 80%), P4 (Yield: 70%), P5 (Yield: 85%), P6 (Yield: 70%), P7 (Yield: 35%), P8 (80%) ^{1}H NMR spectra of: myristoylated tryptophan glucosamine succinate tetra aspartic acid (MWGSD-A_4) in (500 MHz, DMSO-D_6).

^{1}H NMR (500 MHz, DMSO) δ 12.63 (s, 8H), 10.83 – 10.69 (m, 1H), 8.33 – 8.12 (m, 4H), 8.03 (d, 1H), 7.93 (d, 1H), 7.57 (dd, *J* = 31.3, 8.7 Hz, 1H), 7.30 (d, *J* = 7.6 Hz, 1H), 7.10 (dd, *J* = 8.7, 7.6 Hz, 1H), 7.03 (dd, 1H), 6.97 (dd, *J* = 7.7 Hz, 1H), 6.05 – 5.71 (m, 1H), 5.34 – 5.15 (m, 1H), 5.14 – 4.97 (m, 1H), 4.97 – 4.79 (m, 1H), 4.56 – 4.43 (m, 4H), 4.19 – 3.99 (m, 4H), 3.40 – 3.34 (m, 2H), 2.97 – 2.77 (m, 2H), 2.70 – 2.61 (m, 4H), 2.60 – 2.54 (m, 4H), 2.48 – 2.34 (m, 12H), 2.33 – 2.23 (m, 2H), 2.01 (t, *J* = 8.4 Hz, 2H), 1.39 – 1.30 (m, 2H), 1.26 – 1.14 (m, 20H), 0.86 (t, *J* = 6.7 Hz, 3H).

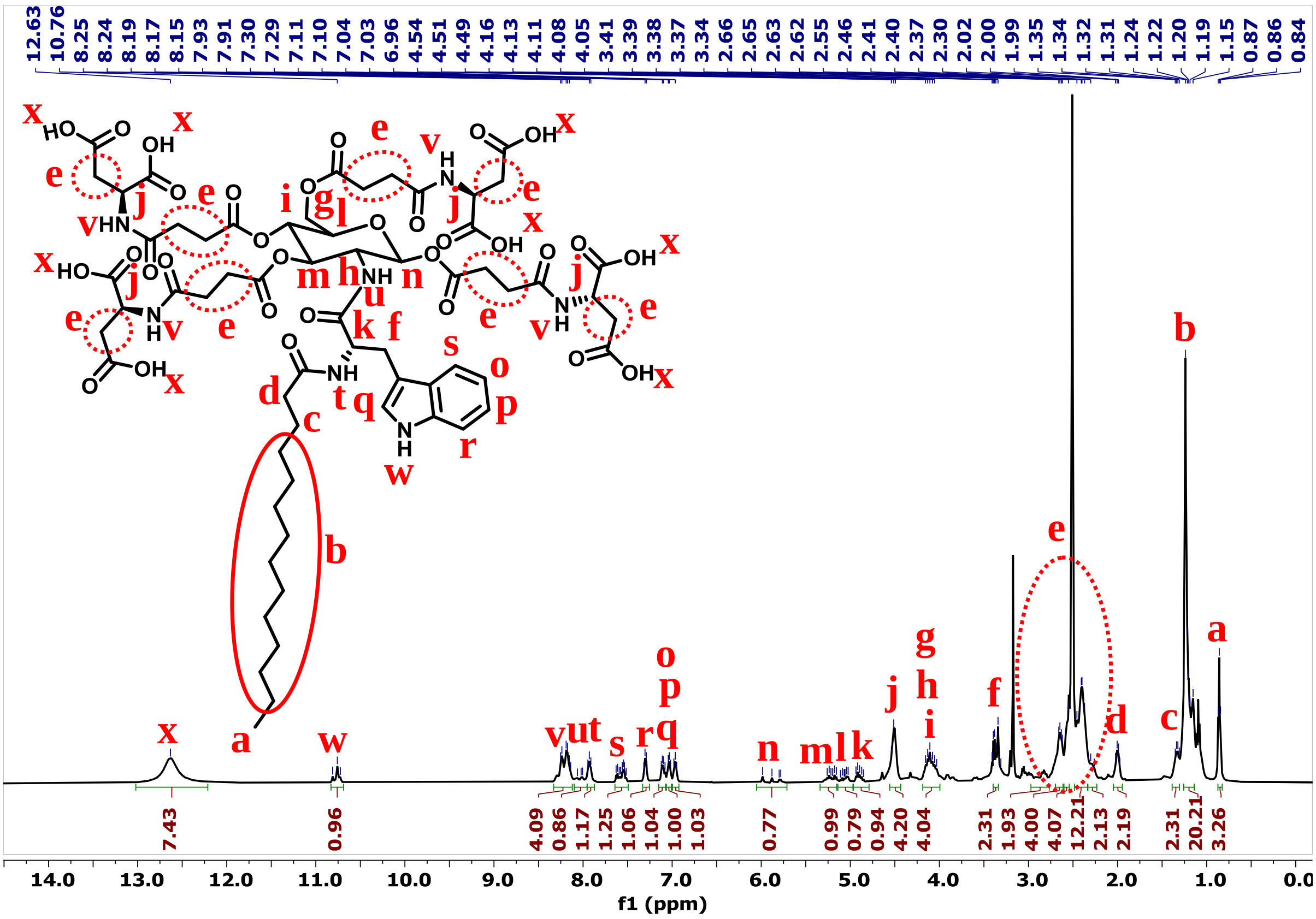

Figure S9: ^{1}H NMR of myr-tryptophan glucosamine tetra succinate tetra aspartic acid (**MWGSD-A$_4$**).

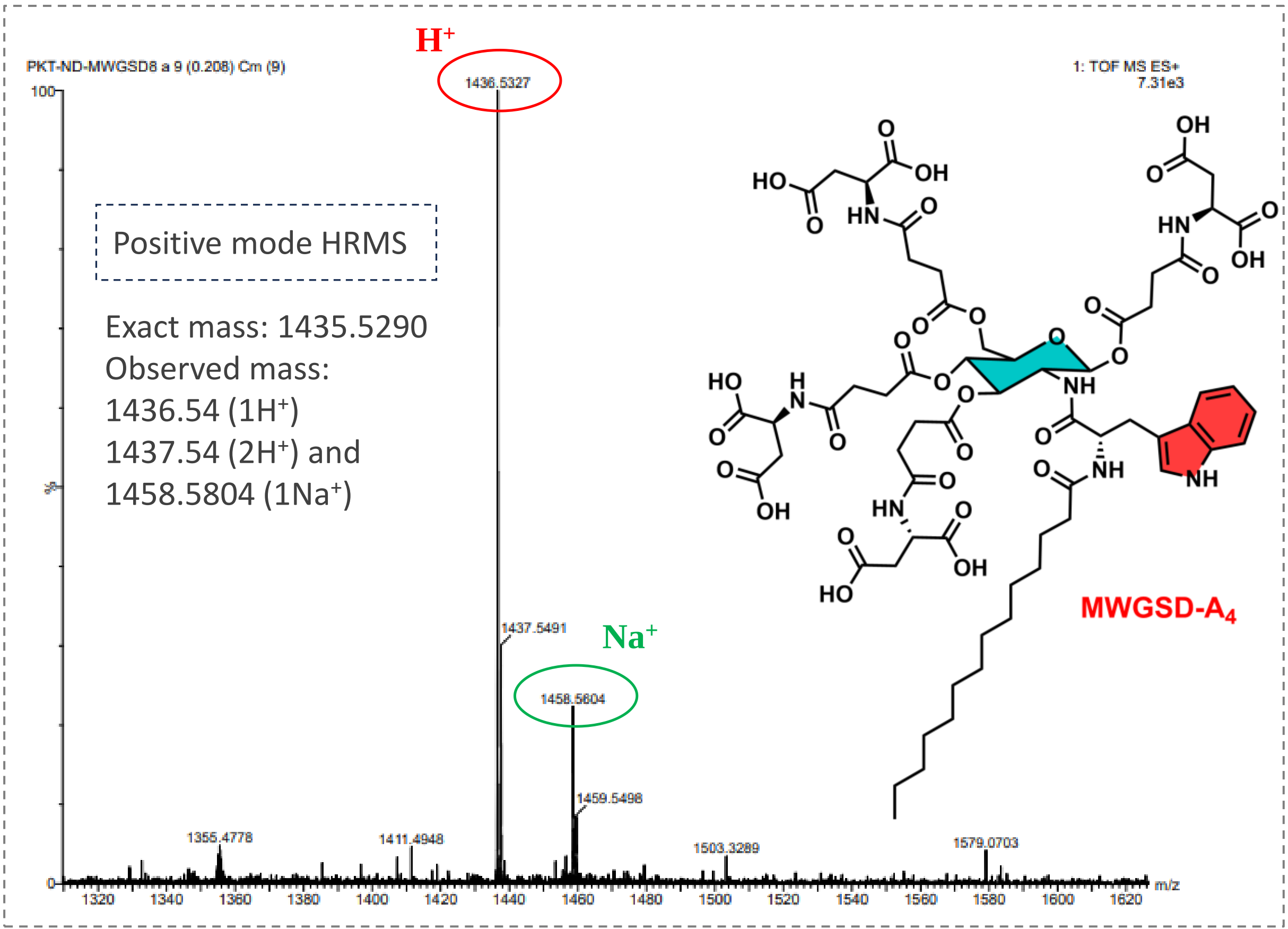

Figure S10: HRMS of MWGSD-A_4.

Synthesis of Myr- Glucosamine Succinate Tetra Aspartic Acid (MGSD-A_4): P1 (Yield: 85%), P2 (Yield: 80%), P3 (Yield: 80%), P9 (Yield: 80%), P10 (Yield: 85%), P11 (Yield: 75%), P12 (Yield: 40%) P13 (80%) ^{1}H NMR spectra of myristoylated glucosamine succinate tetra aspartic acid (MGSD-A_4): (500 MHz, DMSO-D_6).

^{1}H NMR (500 MHz, DMSO) δ 12.55 (s, 8H), 8.31 – 8.14 (m, 4H), 7.81 (d, J = 9.2 Hz, 1H), 5.72 (d, J = 8.8 Hz, 1H), 5.26 – 5.16 (m, 1H), 4.94 – 4.85 (m, 1H), 4.56 – 4.46 (m, 4H), 4.18 – 4.09 (m, 1H), 4.06 – 3.91 (m, 3H), 2.71 – 2.61 (m, 4H), 2.60 – 2.50 (m, 17H), 2.48 – 2.38 (m, 8H), 2.41 – 2.28 (m, 9H), 2.01 (t, 2H), 1.42 (t, J = 7.3 Hz, 2H), 1.22 (d, J = 10.5 Hz, 20H), 0.86 (t, J = 6.8 Hz, 3H).

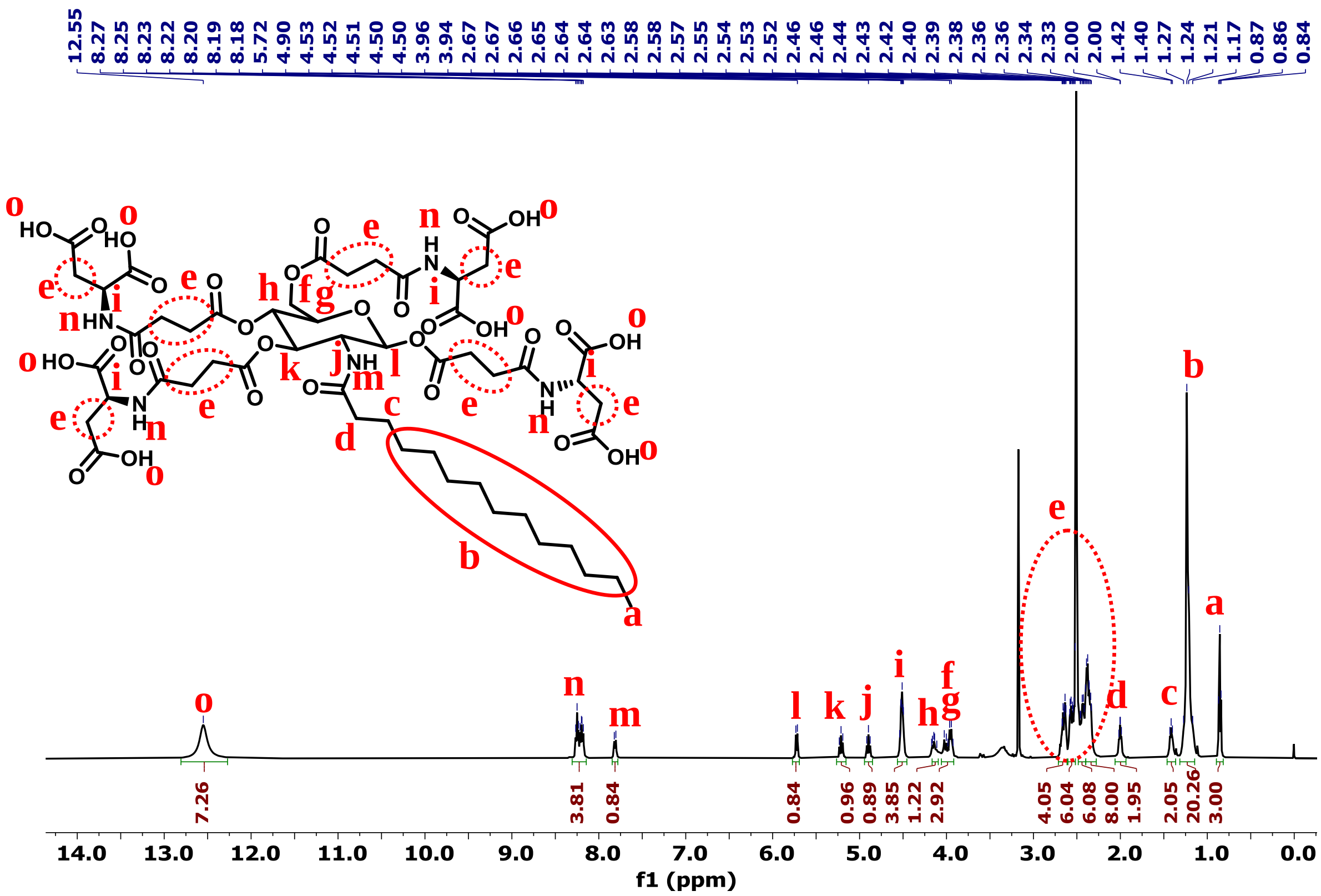

Figure S11: ^{1}H NMR of MGSD-A_4

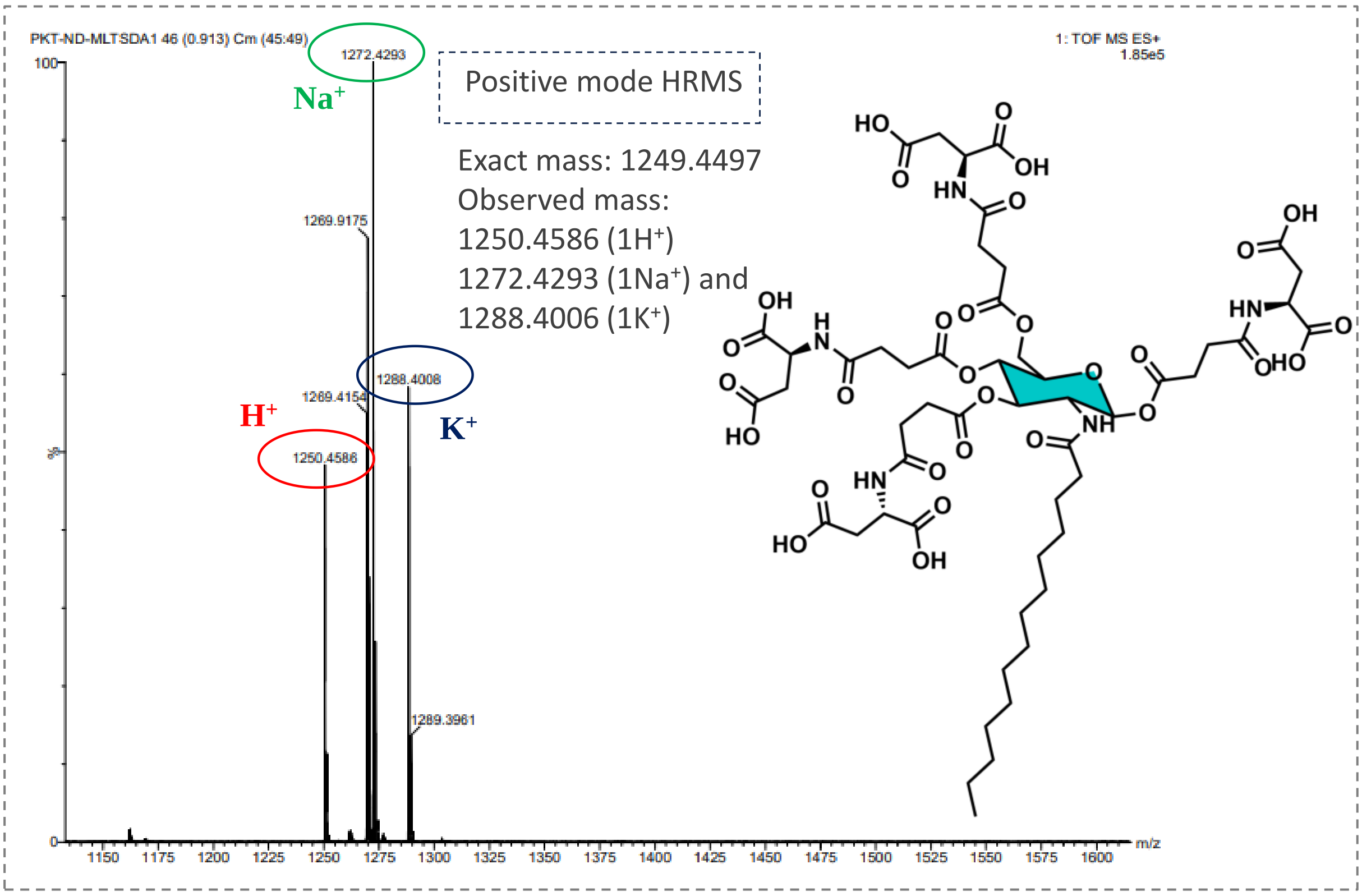

Figure S12: HRMS of MGSD-A_4.

Purify the compounds by HPLC:

Reverse-phase HPLC was performed using a C-18 column on a Waters 1525 binary HPLC pump and a PDA detector. A stock solution of 2 mg/ml of MWGSD-A_4 was prepared in water. 20 µL of the solution was injected and eluted with a linear gradient of 10% MeCN to 90% MeCN over 30 minutes at a flow rate of 1 mL/min.

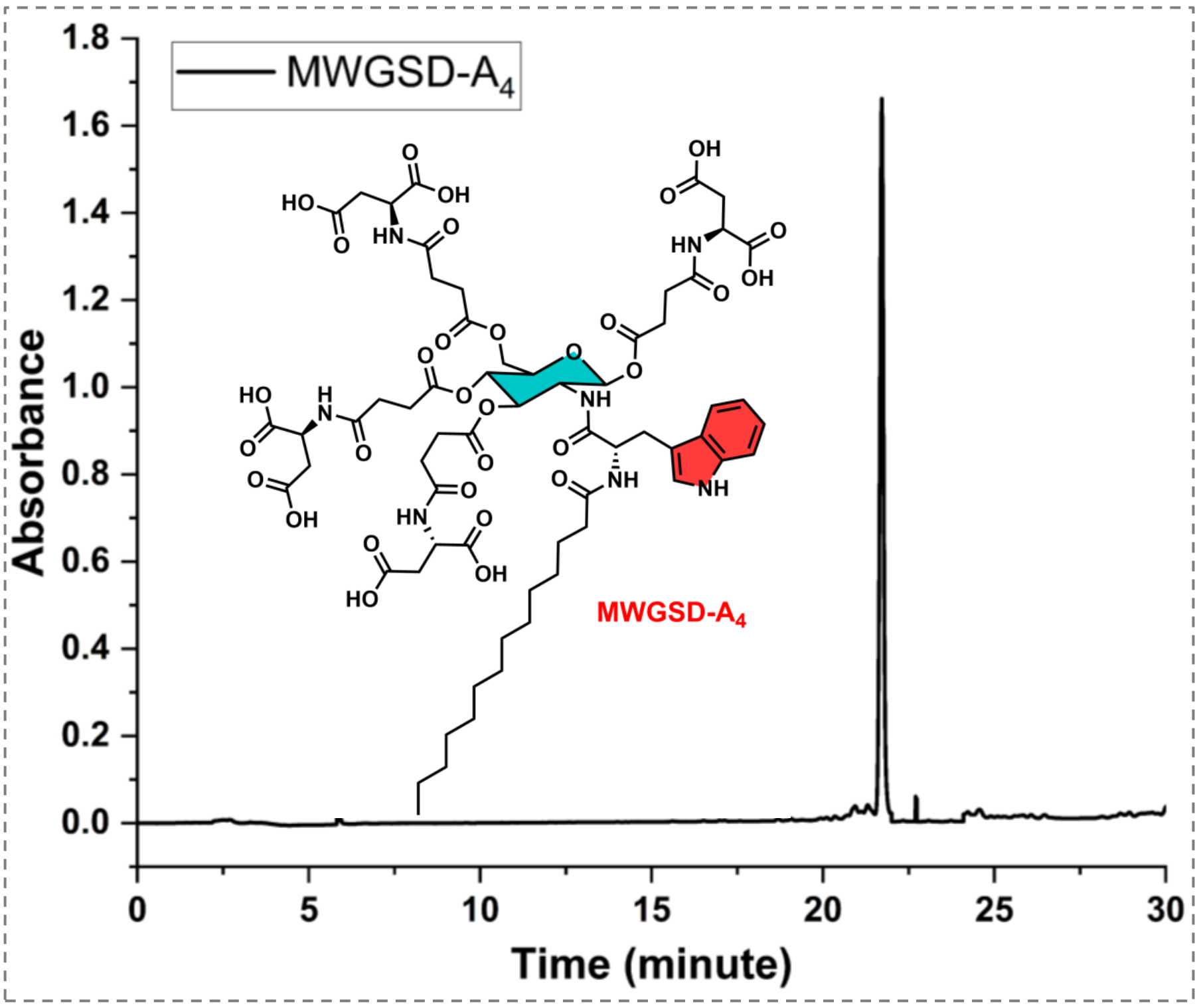

Figure S13: HPLC of MWGSD-A_4 (92 % Pure).

Nile Red (NR) Fluorescence

NR, a solvatochromic dye, was used to assess the critical aggregation concentration (CAC). 2 µM Nile Red was added to 1 mL of 20 mM MES buffer (pH 5.5) at 25 ^{o}C. Fluorescence emission spectra were collected from 550 to 700 nm following excitation at 534 nm. Next, lipidated glycopeptide mimics (MWG, MWGS-A_4, MWGSD-A_4, and MGSD-A_4) were added to the solution, and the NR emission spectra were recorded after equilibration. The experiments were further performed at various concentrations of lipidated glycopeptide mimics (LGMs). The fluorescence measurements were conducted using a Hitachi F-7000 spectrofluorometer (Japan) with a 1 cm path length quartz cuvette. Both excitation and emission slits were set to 5 nm bandpass to ensure consistent detection parameters throughout the experiments.

Dynamic Light Scattering (DLS)

The hydrodynamic diameter of the samples was measured using a Malvern Zetasizer Nano ZS (UK) equipped with a 633 nm He–Ne laser. 100 µM of samples (MWG, MWGS-A_4, MWGSD-A_4, and MGSD-A_4) were prepared in 20 mM MES buffer (pH 5.5), and DLS measurements were performed at 25 °C. The samples were polydisperse, and DLS experiments were performed three times to estimate the particle size.

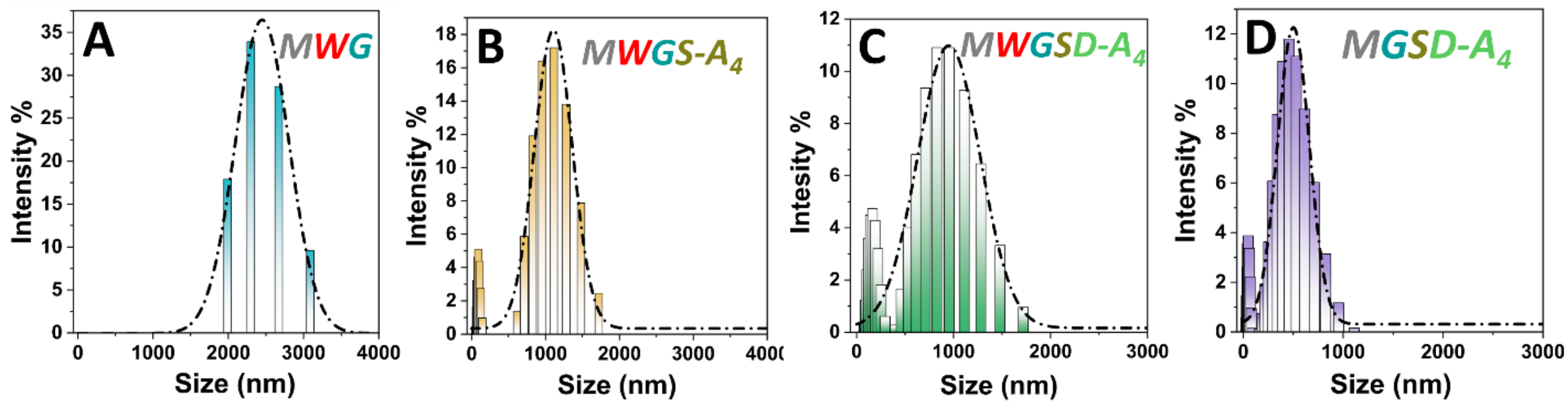

Figure S14: DLS profile of 100 µM glycolipid-based amphiphiles. (All the studies were conducted in 20 mM MES buffer (pH 5.5).

Confocal microscopic studies to analyse coacervation

100 µM solutions of LGMs (MWG, MWGS-A_4, MWGSD-A_4, and MGSD-A_4) were prepared in 20 mM MES buffer at pH 5.5 from 100 mM DMSO stock. The solution was incubated at room temperature for 10 minutes for equilibration. Next, the fluorophore (hydrophilic dye: fluorescein disodium; hydrophobic dye: Nile Red) was added and incubated for 30 minutes. The dye concentration was 0.5 µM. After dye incorporation, the sample was drop-cast onto a clean glass slide and gently covered with a coverslip to ensure a uniform surface for imaging. Confocal laser scanning microscopy (CLSM) was performed using a Leica SP8 system (Leica Microsystems, Wetzlar, Germany) equipped with an HC PL APO 63x/1.40 oil immersion objective. Fluorescence excitation was carried out using 488 nm and 552 nm laser lines, appropriate for the dyes used. The DIC images were acquired without the fluorophore. The effects of pH, NaCl, and urea were determined using 100 µM MWGSD-A_4, with Nile Red as the fluorophore.

FRAP measurement

The FRAP experiment was performed using 100 µM MWGSD-A_4 in a pH 5.5 buffer with an Olympus IX83 microscope. The dispersion of MWGSD-A_4 was treated with sulforhodamine B for 30 minutes. Pre-bleaching images were acquired with an imaging power set at 4%. Subsequently, we selected a whole droplet as a region of interest (ROI) at a specific focus plane and bleached it at 25% power using the 552 nm laser. The fluorescence intensity of the ROI over the duration of the experiments and the fluorescence recovery were determined by measuring the ROI's fluorescence during the post-bleach sequence. Three or more measurements were conducted, and the fluorescence intensities of the ROIs (extracted using ImageJ) were plotted.

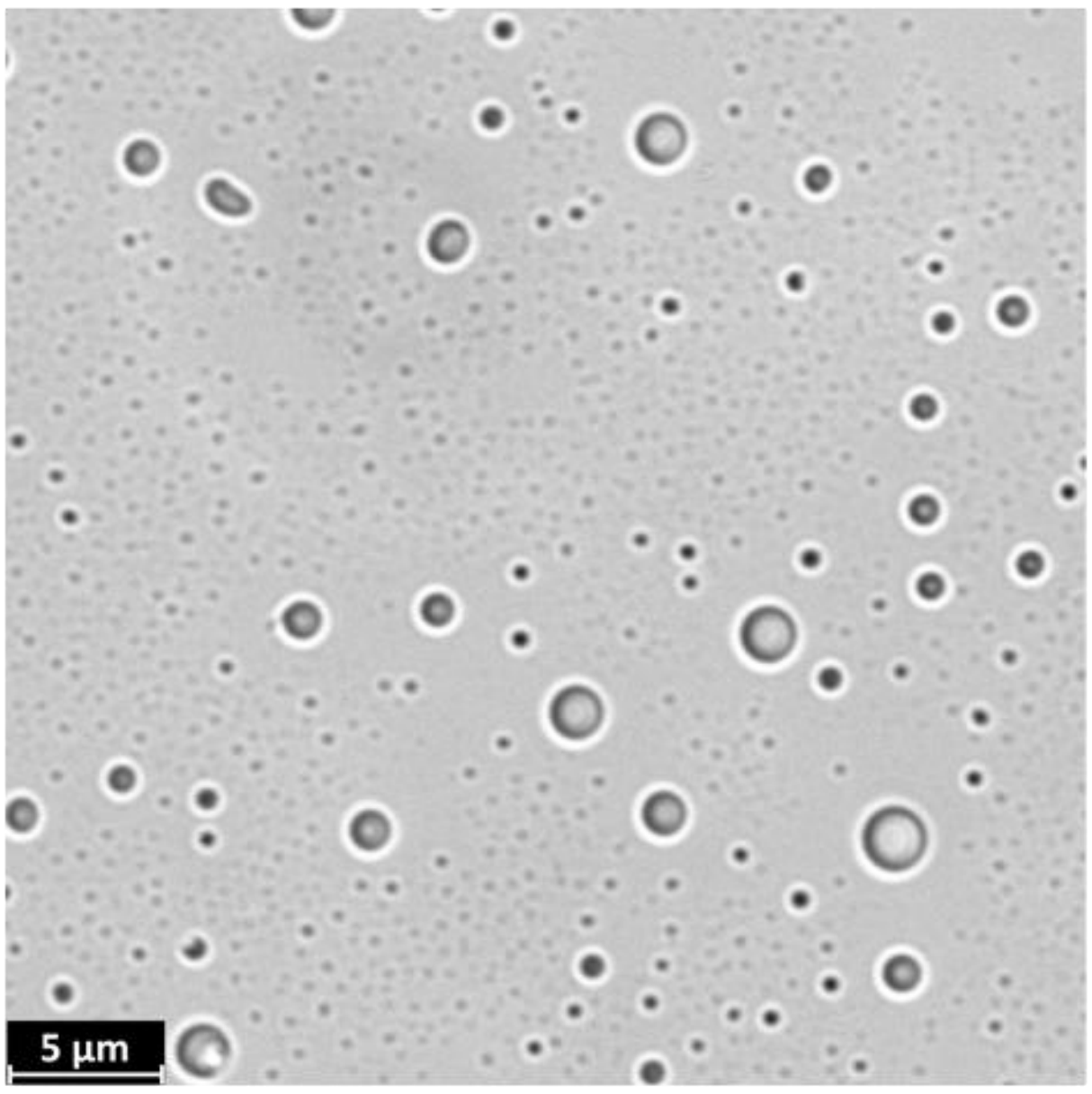

Figure S15: DIC images of MWGSD-A_4 coacervate at 100 µM.

Cryo-TEM (Transmission Electron Microscopy)

Cryogenic transmission electron microscopy (Cryo-TEM) was employed to examine the morphology of MWGSD-A_4 using a JEM-2100 Plus microscope (JEOL). 100 µM solution of MWGSD-A_4 was prepared in 20 mM MES, pH 5.5 buffer and equilibrated for 15 minutes. Afterwards, a small volume (2 µL) was drop-cast onto a TEM grid and allowed to adsorb for 45 seconds. The grid was then rapidly vitrified by plunge-freezing, and imaging was performed under cryogenic conditions.

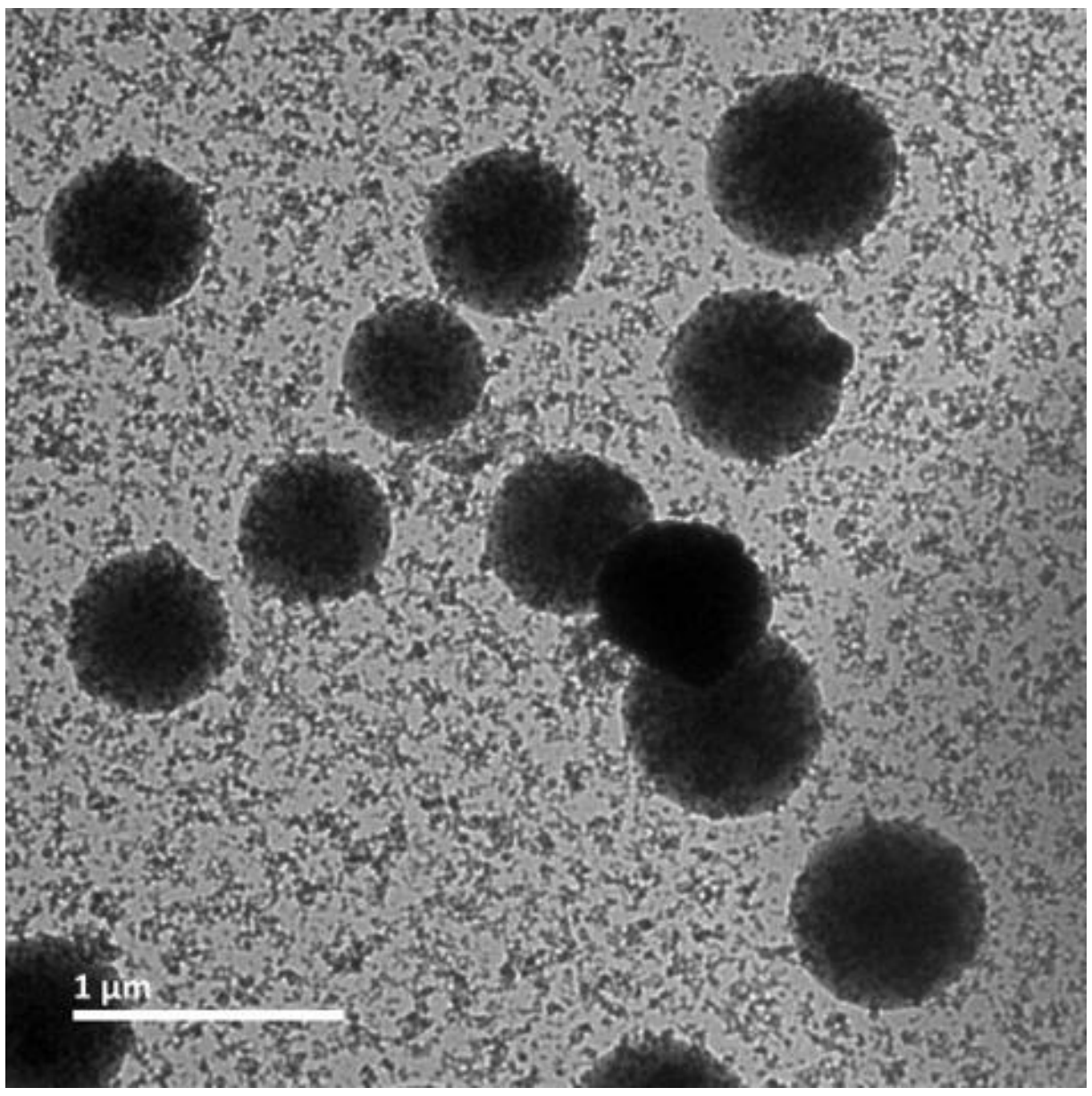

Figure S16: Cryo TEM image of **MWGSD-A_4** at 100 µM.

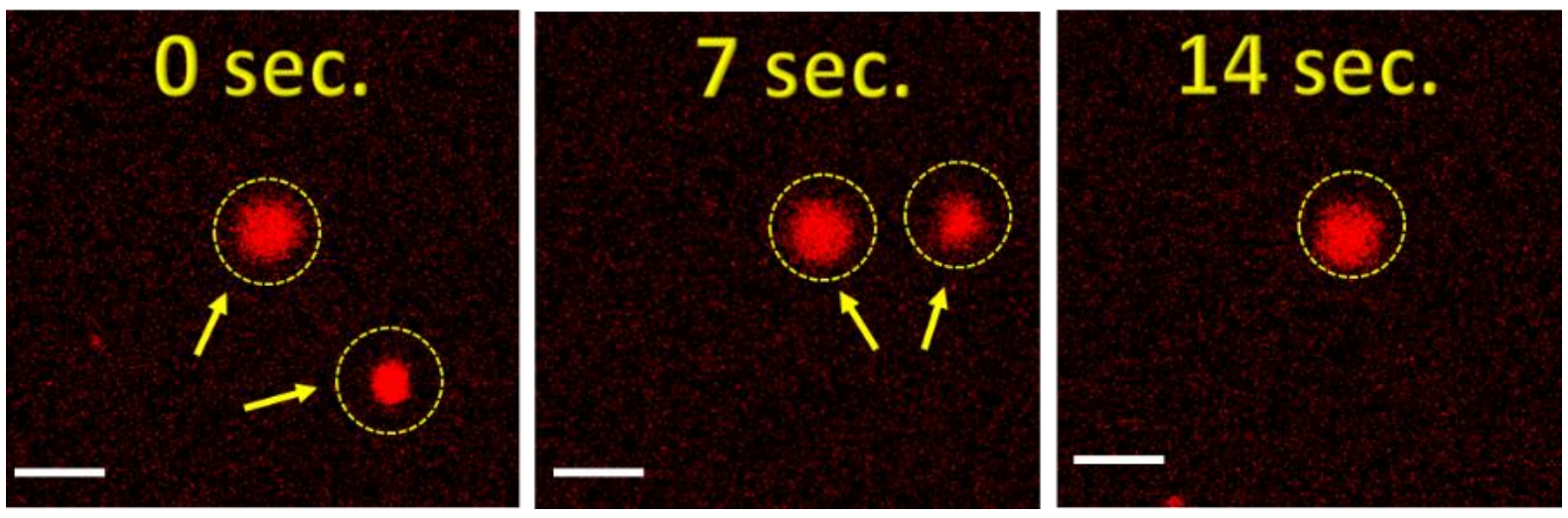

Figure S17: Coalescence image of **MWGSD-A_4** at 100 µM. The scale bar is 2 µm.

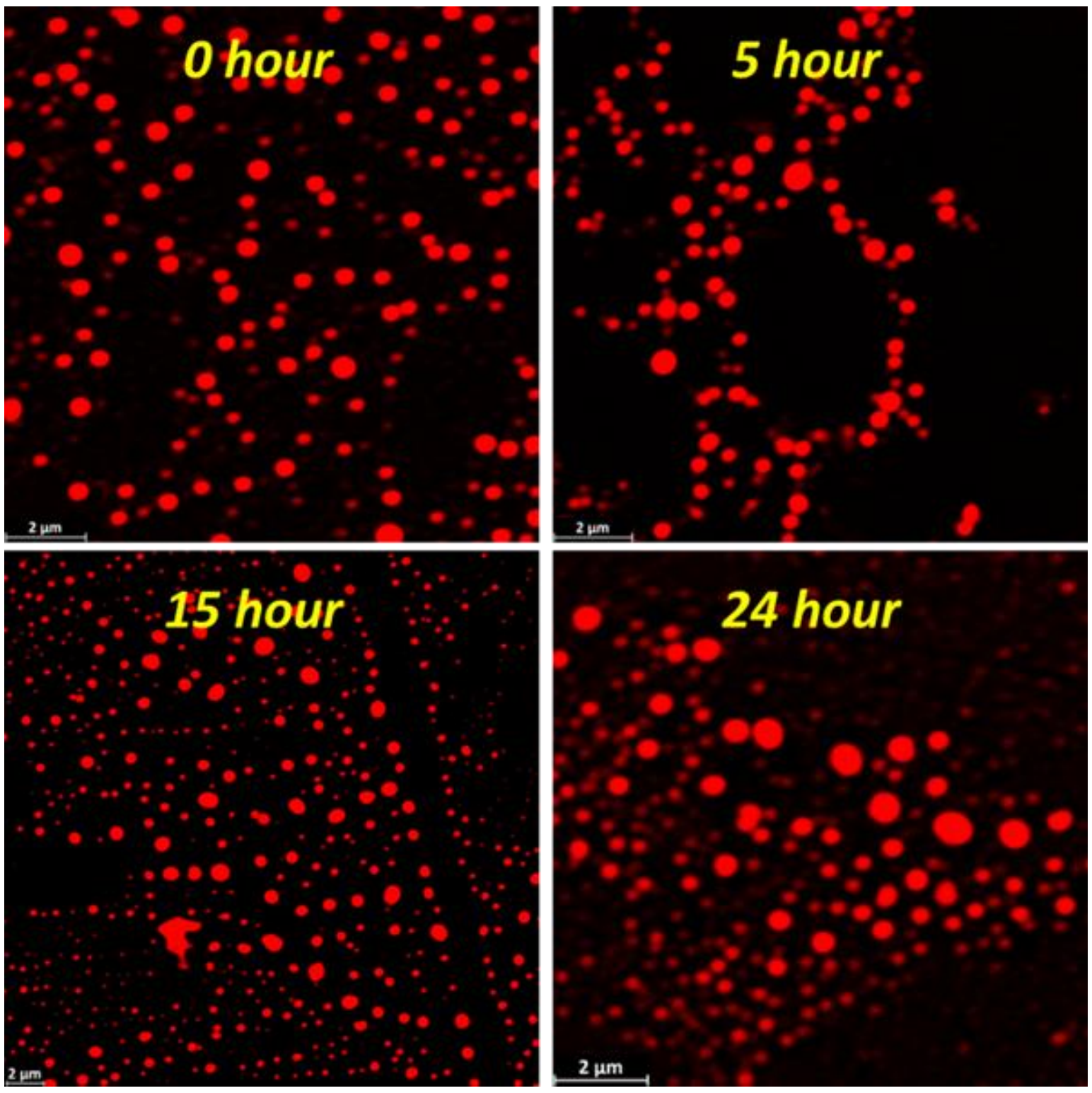

Figure S18: Time-dependent imaging of **MWGSD-A_4** at 100 µM. The scale bar is 2 µm.

Preparation of FITC-Labelled Insulin and BSA

5 mg/mL of the protein was dissolved in 100 mM $NaHCO_3$-Na_2CO_3 buffer solution (pH 9.0). FITC (2 mg) in 50 µL of DMSO was added to the protein solution at 25 ^{o}C and kept overnight under stirring at dark. After that, the reaction mixture was dialyzed (dialysis membrane; cutoff: 6-8 KDa) against a cold buffer solution, periodically replenishing with buffer, until the unreacted FITC's green emission was completely reduced. Finally, the protein solution was dialyzed against cold Milli-Q water to remove the salts. FITC-tagged protein was obtained by lyophilising the mixture.

Aggregation of GFP-expressing Lentiviral particles and Influenza particles

To assess the effect of MWGSD-A_4 coacervate in viral aggregation/sequestration GFP expressing lentivirus (GFP-LV) was used. The pHIV-Luc-ZsGreen Plasmid and HEK293T cells (BEI Resources, USA) were used to generate replication-inefficient GFP-expressing lentiviral particles. The infectious titre of the harvested lentiviral particles was determined using the standard 50% $TCID_{50}$ assay. MWGSD-A_4 coacervates were incubated with GFP-lentiviral particles for 12 h, and confocal microscopy was performed to investigate the sequestration.

A 488 nm laser was used to excite the GFP-lentiviral particles. The MWGSD-A_4 coacervates were also labelled with Nile red to investigate the colocalization.

Glycolipids (MWG, MWGS-A_4, MGSD-A_4, and MWGSD-A_4) were incubated with influenza A/Puerto Rico/8/1934 (H1N1) virus (BEI Resources, USA) and incubated for for 12 h. The OD at 400 nm was measured in Epoch2 BioTek microplate reader.

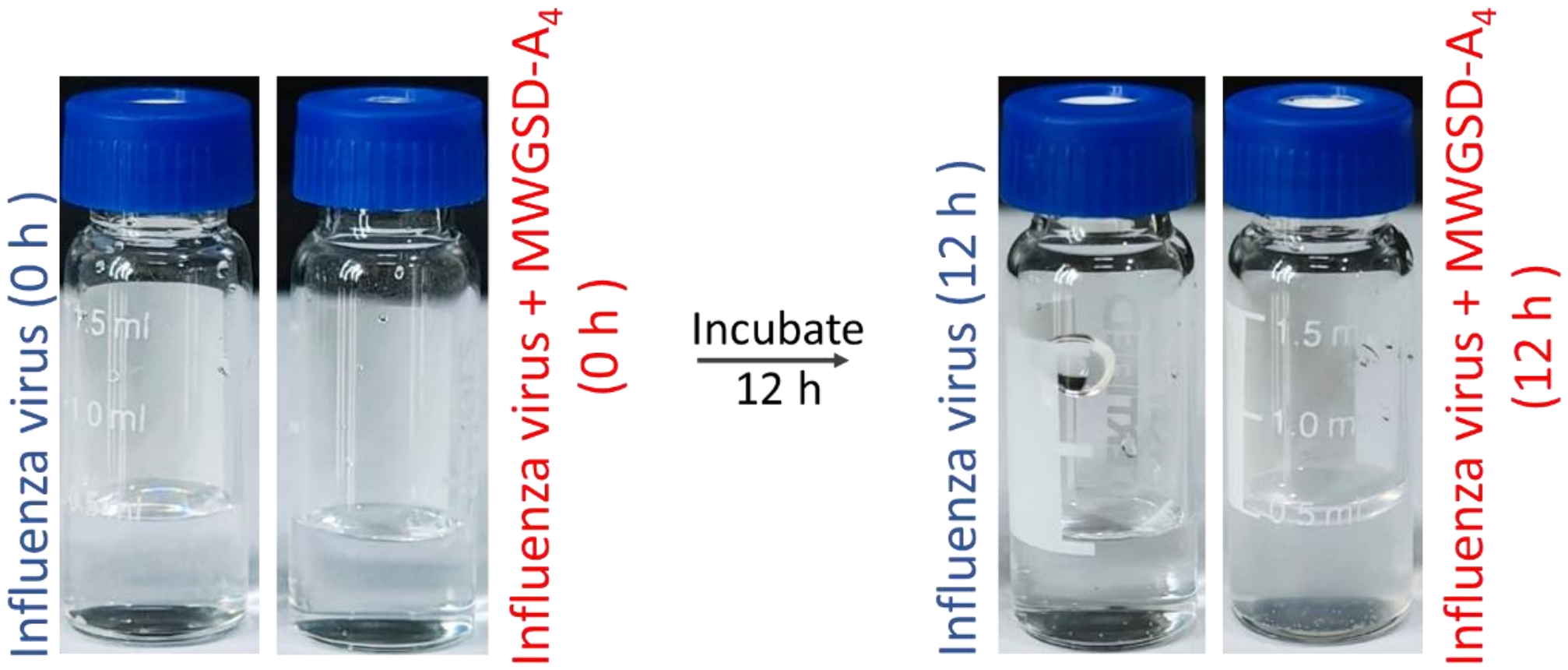

Figure S19: Incubation of the MWGSD-A_4 coacervate with influenza virus.

Influenza Virus entry assay

PA-2A-Swap-Nluc (PASTN) reporter virus based on the strain influenza A/WSN/1933 (H1N1) was used in a Nano-Luciferase reporter assay to determine the luciferase activity of this reporter virus, as previously described by Tran et al. Deciphering the antiviral activity of a compound using a nanoluciferase-based, highly sensitive influenza A reporter virus was previously described.[1, 2] Briefly, MDCK cells were seeded in a 96-well plate and treated with increasing concentrations of the compound for 1h at 37°C. At the same time, the influenza viruses at a multiplicity of infection (MOI) of 0.01 were also treated with different concentrations of MWGSD-A_4 for 1h at RT. Thereafter, the cells were infected in triplicate with a treated inoculum of the nanoluciferase influenza A reporter virus (PASTN) for 1h. Thereafter, the cells were replenished with the same concentration of the MWGSD-A_4. The infected cells were harvested at 8hpi, and the viral luciferase activity was measured using the Nano-Glo luciferase assay system (Promega). We performed these experiments independently three times. Luminescence was detected using a Luminometer (Glomax 20/20, Promega), a direct measure of viral entry into cells.

MTT assay

MDCK cells were seeded in respective 96-well plates, followed by a dose-dependent increase in MWGSD-A_4 concentration for 24 h at 37 °C in 5% CO_2. Thereafter, the media was aspirated, and 100 µL of MTT reagent (5 mg/mL, SRL) in phosphate-buffered saline (PBS) was added to the cells, and incubated for 3h at 37 °C. Subsequently, the MTT reagent was removed, and the formazan crystals thus formed were dissolved in 100 µL of dimethyl sulfoxide (DMSO, Sigma). The absorbance of the DMSO-dissolved formazan crystal suspension was determined at 595 nm using an Epoch 2 microplate reader (BioTek Instruments). The percentages of metabolically active cells thus measured were compared with the percentage of DMSO-treated control cells. The assay was repeated in triplicate and independently three times.

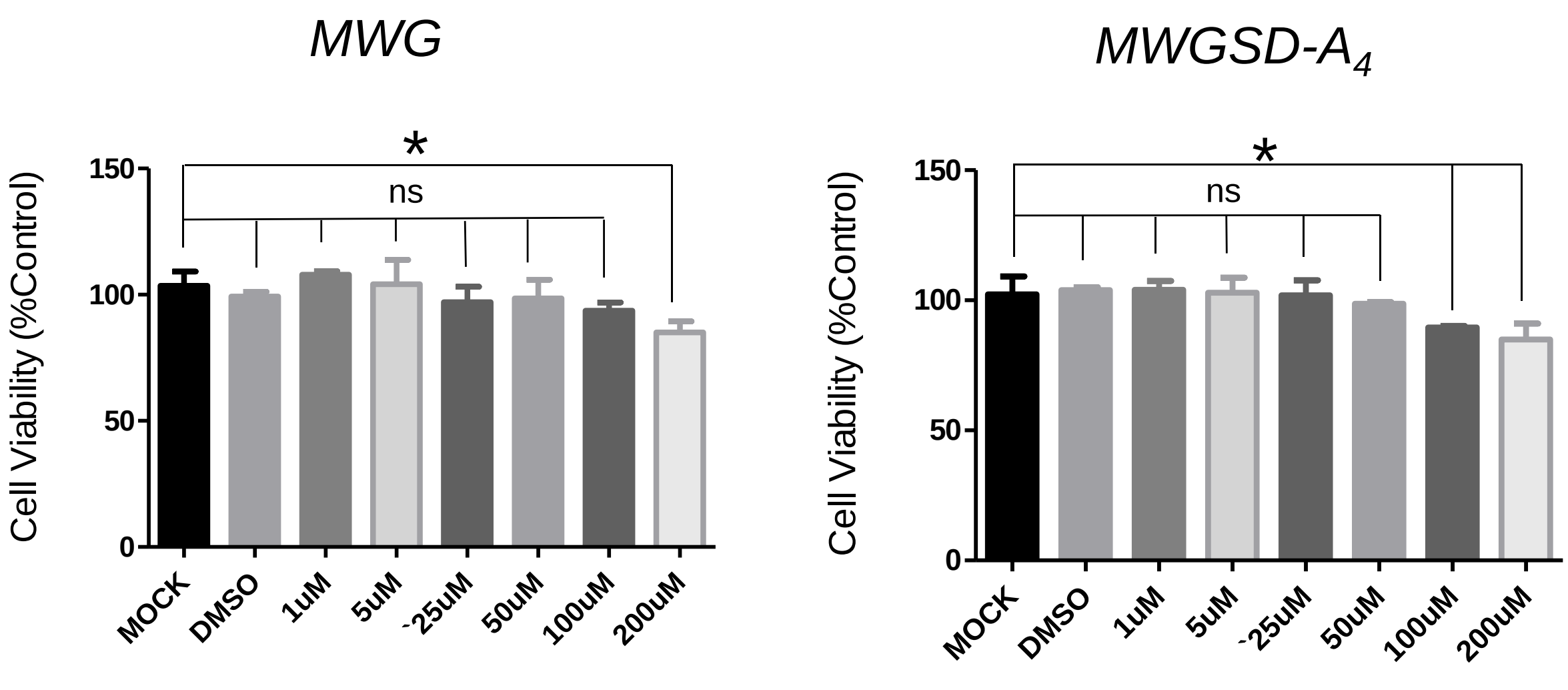

Figure S20: Cell viability of MWG and MWGSD-A_4 against the MDCK cell line.

Hemolysis assay

The hemocompatibility of the compounds was evaluated using a standard hemolysis assay with freshly isolated chicken erythrocytes (cRBC). Briefly, fresh chicken blood was collected in Alsever's solution to prevent clotting, and cRBCs were separated by centrifugation at 500 rpm for 10 min, followed by washing with sterile 1× PBS (pH 7.4). Finally, the pellet was resuspended in 1××x PBS to make 5% (v/v) suspension. 100 µL of RBC solutions and 900 µL PBS were kept in sample tubes. Various concentrations of lipidated glycopeptide mimics were added to each tube and incubated at 37 ^{o}C. The sample with no compound was used as the negative control, and 1% Triton X-100-treated samples were used as the positive

control. After 24 h, the sample was centrifuged for 10 minutes at 3000 rpm. Next, the supernatant was collected, and OD at 540 nm was measured in BioTek Epoch 2 Microplate Spectrophotometer. The percentage of hemolysis was calculated according to the following equation.

$$\% \text{ Hemolysis} = [(A_{sample} - A_{negative\ control})/(A_{positive\ control} - A_{negative\ control})] \times 100$$

Hemagglutination (HA) assays

A standard HA assay was performed using the influenza A/Puerto Rico/8/1934 (H1N1) virus (BEI Resources, USA) at an MOI of 0.1 to examine the ability of glycolipids to interfere with the interaction between the influenza virus and cRBCs following the published protocol.[3] Briefly, two-fold serial dilutions of each compound were prepared in sterile 1x PBS (pH 7.4), and 100 µL of each dilution was added to wells of a 96-well U-bottom plate. An equal volume (100 µL) of freshly prepared cRBCs (0.5% cRBCs suspended in 1x PBS) was then added to each well. Next, 100 µL of the virus was added to each well and incubated at room temperature for 30 min. Well containing PBS without virus served as a positive control, while the virus sample without the coacervate served as a negative control. Wells were examined visually for hemagglutination (characterised by RBC lysis), and wells showing a compact "button" or teardrop formation indicated hemagglutination inhibition.

References:

1. Das Jana, I.; Kumbhakar, P.; Banerjee, S.; Gowda, C. C.; Kedia, N.; Kuila, S. K.; Banerjee, S.; Das, N. C.; Das, A. K.; Manna, I.; Tiwary, C. S.; Mondal, A., *ACS Applied Nano Materials* **2021,** *4* (1), 352-362. DOI 10.1021/acsanm.0c02713.
2. Tran, V.; Moser, L. A.; Poole, D. S.; Mehle, A., *Journal of Virology* **2013,** *87* (24), 13321-9. DOI 10.1128/jvi.02381-13.
3. Yadav, S.; Varma, A.; Muralidharan, A. O.; Bhowmick, S.; Mondal, S.; Mallick, A. I., *Gut Pathogens* **2023,** *15* (1), 53. DOI 10.1186/s13099-023-00578-5.
